# Plausible role of INPP4A dysregulation in idiopathic pulmonary fibrosis

**DOI:** 10.14814/phy2.16032

**Published:** 2024-05-08

**Authors:** Meghana Arvind, Bijay Pattnaik, Atish Gheware, Y. S. Prakash, Mousami Srivastava, Anurag Agrawal, Naveen Kumar Bhatraju

**Affiliations:** ^1^ Centre of Excellence for Translational Research In Asthma and Lung diseases (TRIAL) CSIR‐Institute of Genomics and Integrative Biology New Delhi India; ^2^ Academy of Scientific and Innovative Research (AcSIR) Ghaziabad India; ^3^ Department of Pulmonary Critical Care and Sleep Medicine All India Institute of Medical Sciences New Delhi India; ^4^ Division of Pulmonary and Critical Care Medicine, Department of Medicine Washington University in St. Louis St. Louis Missouri USA; ^5^ Department of Anaesthesiology and Perioperative Medicine Mayo Clinic Rochester Minnesota USA; ^6^ Department of Physiology and Biomedical Engineering Mayo Clinic Rochester Minnesota USA; ^7^ Symbiosis Statistical Institute (SSI) Symbiosis International University (SIU) Pune Maharashtra India; ^8^ Trivedi School of Biosciences Ashoka University Sonipat Haryana India

**Keywords:** bleomycin, disease heterogeneity, idiopathic pulmonary fibrosis, INPP4A, transforming growth factor‐β

## Abstract

INPP4A has been shown to be involved in the regulation of cell proliferation and apoptosis of multiple cell types including fibroblasts. Previous reports from our group have demonstrated the role of inositol polyphosphate 4‐phosphatase Type I A (INPP4A) in these functions. Though existing evidences suggest a critical role for INPP4A in the maintenance of lung homeostasis, its role in chronic lung diseases is relatively under explored. In the current study, we made an attempt to understand the regulation of INPP4A in idiopathic pulmonary fibrosis (IPF). Through integration of relevant INPP4A gene expression data from public repositories with our results from in vitro experiments and mouse models, we show that INPP4A is altered in IPF. Interestingly, the direction of the change is dependent both on the disease stage and the region of the lung used. INPP4A was found to be upregulated when analyzed in lung sample representative of the whole lung, but was downregulated in the fibrotic regions of the lung. Similarly, *INPP4A* was found to be high, compared to controls, only in the early stage of the disease. Though the observed increase in *INPP4A* was found to be negatively correlated to physiological indices, FVC, and DL_CO_, of lung function, treatment with anti‐INPP4A antibody worsened the condition in bleomycin treated mice. These contrasting results taken together are suggestive of a nuanced regulation of INPP4A in IPF which is dependent on the disease stage, cellular state and extent of fibrosis in the lung region being analyzed.

## 1 INTRODUCTION

Idiopathic pulmonary fibrosis (IPF) is a chronic, progressive and fibrotic interstitial pneumonia with unknown etiology, frequently occurring in older males. It is the most common form of progressive fibrosing interstitial lung disease (ILD). IPF is a fatal disease with heterogeneous clinical course and poor prognosis. Most of the IPF patients have low survival rates and die within 2–3 years of diagnosis (Travis et al., 2013). The understanding of IPF pathophysiology has been evolving through the years. Initially, IPF was considered as a chronic inflammatory disease, due to the observed association between inflammatory infiltrates and the fibrosis inside IPF lungs, and was treated with immunosuppressive agents. However, multiple clinical trials of immunomodulatory drugs for IPF treatment showed no clinical benefit. Currently, it is thought to be a disease of repeated injuries to the alveolar epithelium aggravating dyspnea ultimately leading to progressively declining lung function (Raghu, 2017). The detrimental effect of immunosuppressive therapies on IPF patients and the effectiveness of recent anti‐fibrotic drugs in slowing down the disease progression support the current view of epithelial injury as the disease initiator. IPF has no known cure till now and therefore identification of clinically relevant disease biomarkers and effective therapeutic targets remains an active area of research (Somogyi et al., 2019). PI3K/AKT signaling, known for its role in the regulation of key fibrotic processes such as alveolar cell apoptosis, epithelial‐mesenchymal transition (EMT) and myofibroblast differentiation, has attracted significant attention in the recent years as a promising therapeutic target in IPF. Based on the strong evidence supporting the involvement of PI3K/AKT signaling in fibrotic processes, PI3K inhibitors are currently under clinical evaluation for the treatment of IPF and myelofibrosis (Wang et al., 2022; Zhao et al., 2022).

Inositol polyphosphate‐4‐phosphatase type I A (INPP4A) is an enzyme belonging to the family of lipid phosphatases that converts phosphatidyl inositol (3,4)‐bisphosphate (PI(3,4)P2) to phosphatidyl inositol 3‐phosphate (PI(3)P) (Norris & Majerus, 1994). By altering the levels of phosphoinositides at the cell membrane, INPP4A negatively regulates the phosphatidylinositol 3‐kinase (PI3K) signaling pathway (Ivetac et al., 2009; Munday et al., 1999; Norris et al., 1997). It's role has also been demonstrated in critical cellular processes such as growth, proliferation and apoptosis (Ivetac et al., 2009; Vyas et al., 2000; Wang et al., 2018). INPP4A is also known to shuttle to nucleus (Chaudhuri et al., 2018) and be secreted to the extracellular space in free form and in extracellular vesicles (Khanna et al., 2019). The earliest report for the role of INPP4A in the lung showed the genetic association of INPP4A with atopic asthma (Sharma et al., 2008). Functional studies have highlighted the importance of INPP4A in maintaining normal lung structure, and loss of cellular and secretory INPP4A in naïve mice developed spontaneous airway hyperresponsiveness and airway remodeling. Previous studies where depletion of cellular or secretory INPP4A was shown to aggravate allergic airway inflammation (AAI) phenotype also suggests a protective role for INPP4A in AAI (Aich, Mabalirajan, Ahmad, Khanna, et al., 2012; Khanna et al., 2019). Shuttling of INPP4A to nucleus has been shown to be associated with functions of cell cycle regulation, proliferation and apoptosis (Chaudhuri et al., 2018).

INPP4A has been shown to regulate cell proliferation in various cell types including fibroblast cells (Ivetac et al., 2009; Khanna et al., 2019; Vyas et al., 2000). However, the role of this enzyme in human fibrotic lung disorders has not been investigated. The observation that intraperitoneal treatment with anti‐INPP4A antibody increased subepithelial fibroblast proliferation and collagen deposition in both naïve mice and mice with AAI provided a strong rationale to investigate the role of INPP4A in the pathogenesis of pulmonary fibrosis (PF) (Khanna et al., 2019). This hypothesis was further supported by the role of INPP4A in epithelial fibroblast crosstalk evident by in vitro studies where neutralization of INPP4A in the conditioned media of bronchial epithelial cells when added to fibroblasts increased their proliferation (Khanna et al., 2019). In the current study, we made an attempt to understand the regulation of INPP4A in idiopathic pulmonary fibrosis (IPF). Here, we adopted a hybrid approach, wherein we integrated the relevant INPP4A gene expression data from public repositories with our experimental data from mouse models, to gain insight into the spatiotemporal dynamics of the INPP4A in IPF. Some of the findings of this study have been reported earlier in the form of an abstract (Arvind et al., 2021).

## 2 MATERIALS AND METHODS

### 2.1 Clinical material

Written informed consent was obtained from all patients. Lung samples were obtained incidental to patient thoracic surgery at Mayo Clinic Rochester via IRB‐approved protocols that allows for recording of all relevant and available patient data including demographics, pulmonary disease status, PFTs, imaging, co‐morbidities, medications etc. Medical data were recorded, patient identifiers were deleted and samples assigned unique numbers for storage. Patient consent was obtained during pre‐surgical evaluation (Pulmonary, Oncology, or Thoracic Surgery Clinics). All potential patients were reviewed and consented prior to surgery in non‐threatening environs.

### 2.2 Development of bleomycin model of pulmonary fibrosis in mice

Eighteen months old male C57BL/6 mice were maintained at CSIR‐Institute of Genomics and Integrative Biology (IGIB), Delhi, India. The institutional animal ethics committee had approved all protocols used. The animals were kept in conditions of ad libitum feeding, 12 h of light and 12 h of darkness, and a temperature of 20–25°C. Induction of pulmonary fibrosis was performed by instillation of single dose (3.5 U/kg) of bleomycin sulphate (Toronto Research chemicals) intratracheally. Vehicle alone was given to control groups. Mice were sacrificed 21 days after instillation. Mice were anesthetized with i.p. injections of 50 mg/kg xylazine and 100 mg/kg of pentobarbital, lungs were lavaged thrice with 0.4 mL PBS for collection of BAL fluid. After collection and perfusion of BAL fluid, collection of the right lobes of lungs was done for RNA and protein assays and the left lobes were fixed in 10% formalin followed by paraffin embedment. All studies were normally conducted at least twice as separate trials unless otherwise stated.

### 2.3 Histopathology and lung elastance measurement

For qualitative estimation of inflammation, hematoxylin and eosin staining was performed. Fibrosis was estimated using Masson's and Trichrome Staining as described earlier (Ahmad et al., 2014). Measurement of respiratory elastance was performed using computer‐controlled instrument based on invasive airway mechanics measurement (FlexiVent™; SCIREQ, Montreal, Canada) similar to the one described previously (Ahmad et al., 2014). Briefly, post treatment i.p. injections of 50 mg/kg xylazine and 100 mg/kg of pentobarbital were used to anesthetize mice followed by using an 18‐gauge cannula for tracheal intubation. Mice lungs were then provided mechanical ventilation with the use of a computer‐controlled respirator. Respiratory mechanics parameters particularly respiratory system elastance (ERS = 1/compliance) was measured.

### 2.4 INPP4A antibody treatment in vivo

For INPP4A antibody treatment in mice with PF, anti‐INPP4A antibody was utilized (Elabscience E‐AB‐62798). Isotype antibody was used as control (Elabscience). In the murine model of bleomycin induced pulmonary fibrosis, the anti‐INPP4A antibody (1.5 mg/kg body weight) and isotype antibody (1.5 mg/kg body weight) were administered in single doses via the intraperitoneal route in a volume of 200 μL per dose on Days 15, 17, and 19 after which respiratory lung elastance was measured on Day 21 and mice were sacrificed (See Figure 7b). Mice groups used were PBS (saline controls), Bleo (bleomycin treated mice), Bleo /Isotype Ab (Isotype Antibody given to mice with bleomycin induced PF). Bleo/INPP4A Ab (INPP4A antibody given to mice with bleomycin induced PF). For experiments in vivo, littermates were randomly assigned to different experimental groups and each group contained at least five mice unless otherwise stated. Five micrometer thick lung sections were then utilized for immunostaining and other histological analysis. The details of antibodies and key reagents used in the current study are provided in Table 1.

**TABLE 1:** Information about key resources used.

Chemicals			
Serial number	Name	Company	Catalogue number
1	HamsF12	Sigma	N‐6760
2	l‐Glutamine	Invitrogen	25030081
3	Bleomycin sulphate	Toronto Research chemicals	B595800
4	Protease inhibitor cocktail	Sigma	p8340
5	DAB		
6	Bicinchoninic acid (BCA)	Sigma	B9643‐1 L
7	Human TGF‐β1 recombinant protein	Thermo Fisher	PHG9204
8	Triton X‐100	Sigma	T9284
9	SuperSignalTM West femto maximum sensitivity substrate	Invitrogen	34095
10	SuperSignalTM West pico PLUS chemiluminescent substrate	Invitrogen	34580
11	DAPI	Invitrogen	P36966
12	Normal goat serum	Jackson ImmunoResearch Laboratories	005–000‐121
Antibodies			
1	INPP4A	Elabscience	E‐AB‐62798
2	INPP4A	Custom, Link Biotech	S10050‐DR
3	Vimentin	Abcam	ab‐8978
4	E‐cadherin	Santa Cruz	
5	Goat anti‐rabbit IgG‐HRP	GeNei™	114038001A
6	Rabbit anti‐mouse IgG—HRP	GeNei™	1140580011730
7	Donkey anti‐rabbit IgG (H + L) highly cross‐adsorbed secondary antibody, alexa fluor 647	Invitrogen	a31573
8	Donkey anti‐rabbit IgG (H + L) highly cross‐adsorbed secondary antibody, alexa fluor 488	Invitrogen	A‐21206
Kits			
1	INPP4A enzyme ELISA kit	Cloud Clone	SEL624Mu
2	Sircoll assay kit	Bicolor	S1000
3	TGF‐β1 ELISA kit	Invitrogen	88–8350

### 2.5 Immunohistochemistry and quantification

Immunohistochemistry was carried out on 5 μm thick lung tissues that were 4% buffered formalin‐fixed and paraffin embedded using INPP4A antibody (custom made, 1:1500) also used previously (Aich, Mabalirajan, Ahmad, Agrawal, & Ghosh, 2012; Chaudhuri et al., 2018; Khanna et al., 2019). HRP conjugated anti‐rabbit secondary antibody (Genei) was used. Briefly, the tissues were subjected to deparaffinization, rehydration and antigen retrieval. Additional staining with isotype‐matched control IgG (Genei) confirmed the specificity of the primary antibody used. Numerous unbiased and random fields covering the entire lung sections were captured for high confidence and their respective numbers are mentioned in the figures. Quantification was done using ImageJ based on a protocol published by Crowe and Yue (2019).

### 2.6 Immunofluorescence

Paraffin embedded lung tissue sections (5 μm thick) were deparaffinized in xylene and rehydrated in series of graded ethanol and then PBS (pH 7.2). Antigen retrieval was performed by five alternate heating (450°C) and cooling (room temperature) cycles of 3 min each. Immunofluorescence staining was performed overnight in anti‐INPP4A (custom antibody from Imginex), 1:1500 dilution in PBS. Post overnight incubation, slides were washed and treated with Alexa fluor labeled secondary antibody (Invitrogen) for an hour. All sections were counterstained with nuclear DAPI stain using mounting media containing DAPI. Imaging was done at 20× either in Nikon Eclipse Ti2 Confocal Microscope and analyzed on the Nikon Elements software or in in Leica SP8 confocal microscope and analyzed in their associated software. The acquired images were analyzed using ImageJ.

### 2.7 Total cell counts in BAL fluid

After performing BAL, estimation of cell counts was done as explained before (Aich, Mabalirajan, Ahmad, Agrawal, & Ghosh, 2012). Briefly, cold PBS was used to collect bronchoalveolar lavage (BAL). Next, separation of BAL cells and supernatant in the collected BAL fluid was carried out using centrifugation. The cell pellets obtained were washed followed by their resuspension in PBS and a part of it was used for counting the total cell number.

### 2.8 ELISA

INPP4A levels in mice bronchoalveolar lavage fluid (BALF) were measured using ELISA (Cloud‐clone Corp) as per the manufacturer's protocol. Total protein content in the sample was used for normalizing INPP4A levels in the sample. TGF‐β1 levels in total lung lysates were estimated using TGF‐β1 ELISA kit (Invitrogen) as per the manufacturer's instructions.

### 2.9 Cell lines and culturing

A549 (Human type II alveolar epithelial cell line) were obtained from ATCC and maintained in HamsF12 media (Sigma) with 10% FBS and supplemented with 1 mM l‐glutamine.

### 2.10 Collagen content measurement

Measurement of soluble collagen levels in mice's total lung lysates was done with Sircoll Assay kit (Bicolor, Carrickfergus) as per the protocol from the manufacturer.

### 2.11 Immunoblotting

Preparation of total lung and cell lysates was done as per manufacturer's protocol (Sigma). Homogenization was done in RIPA buffer (Sigma). Equal lysate amounts were transferred onto PVDF membrane (MDI, India) after being separated on 8%–10% SDS‐polyacrylamide gel. Staining of membranes with Ponceau S (Sigma) was performed for confirming the transfer. After washing off the stain with Tris buffered saline that contained 0.1% Tween 20 (TBST), the membranes were blocked in 5% BSA/TBST. Membranes were further incubated with primary antibodies for INPP4A (custom antibody; previously used in [Aich, Mabalirajan, Ahmad, Agrawal, & Ghosh, 2012; Chaudhuri et al., 2018; Khanna et al., 2019]), β‐actin (Sigma) and vimentin (Abcam), diluted in 3%–5% BSA/TBST incubated overnight. For E‐Cadherin (Santa Cruz) antibody, incubation was preferably done for 36 h in 3% BSA‐TBST. After incubation with primary antibody, washing of membranes was done using TBST buffer. Next, the membranes were incubated with secondary antibodies conjugated with HRP (Bangalore Genei, India) in 5% BSA/TBST for an hour. Post that, washing of membranes was done with TBST buffer. Bands on all blots were detected using enhanced chemiluminescence.

### 2.12 TGF‐β1 induced EMT induction

A549 cells were grown to 50%–60% confluence and were serum starved in 0% FBS media for 24 h. For induction of EMT, treatment with human TGF‐β1 recombinant protein (Thermo Fisher) was given in 1% FBS containing media. Cells were imaged for the induced mesenchymal morphology in phase contrast microscope and collected for immunoblotting experiment after 120 h.

### 2.13 GEO dataset analysis

Previously published microarray or RNA‐Sequencing data relevant to the current study were obtained from Gene Expression Omnibus database (http://www.ncbi.nlm.nih.gov/geo). Twelve datasets in total were used in the current study. The details of the used datasets are presented in Tables 2 and 3. Processed data were downloaded and analyzed for INPP4A gene expression differences between the healthy and IPF samples. For association studies between INPP4A expression and percent predicted forced vital capacity (FVC% predicted) and percent predicted diffusion capacity of the lung for carbon monoxide (DL_CO_% predicted), publicly available data from the Lung Genomics Research Consortium (https://www.lung‐genomics.org/) was used, the methods and data (Feghali‐Bostwick et al., 2007) available in GSE47460. Additionally, single cell transcriptomics data, GSE136831 (Adams et al., 2020), was used to compare the cell type specific INPP4A expression in IPF lungs.

**TABLE 2:** Details of datasets used in Figure 1.

Serial number	Accession number	Platform	IPF patients (*n*)	Controls (*n*)	References
1	GSE32537	Bulk RNA microarray	119	50	Yang et al. (2013)
2	GSE53845	Bulk RNA microarray	40	8	DePianto et al. (2015)
3	GSE124685	Bulk RNA sequencing	49	35	McDonough et al. (2019)
4	GSE199949	Bulk RNA sequencing	26	16	Huang et al. (2022)
5	GSE213001	Bulk RNA sequencing	60	40	Jaffar et al. (2022)
6	GSE134692	Bulk RNA sequencing	36	18	Sivakumar et al. (2019)

**TABLE 3:** Details of datasets used in Figure 5.

Serial number	Accession number	Platform	Region of lung sequenced	IPF patients (*n*)	Controls (*n*)	References
1	GSE169500	Bulk RNA sequencing	Fibroblastic Foci and alveolar septae	20	10	Brereton et al. (2022)
2	GSE213001	Bulk RNA sequencing	Apex and base lung regions	55	39	Jaffar et al. (2022)
3	GSE199949	Bulk RNA sequencing	Central and peripheral lung regions	26	16	Huang et al. (2022)
4	GSE124685	Bulk RNA sequencing	Lobes from random lung regions varying in degree of fibrosis	30	54	McDonough et al. (2019)
5	GSE24206	Bulk RNA sequencing	Samples obtained during diagnostic surgical lung biopsy or during orthotopic lung transplantation surgery	16	6	Meltzer et al. (2011)

### 2.14 Statistical analysis

Gene expression differences between the groups was assessed using either *t*‐test or Mann–Whitney *U*‐test based on the result of Shapiro–Wilk normality test. For multiple comparisons, one‐way ANOVA followed by posthoc tests were used. Tukey's HSD or Dunnett's test were used as posthoc tests where applicable. A *p*‐value cutoff of 0.05 was used to reject the null hypothesis. Data analysis was performed in a minimum of three replicates and experiments were carried out at least three times or otherwise as mentioned in the figure legends. Univariate analysis was performed using Pearson's correlation measures to assess the association between clinical data (FVC, DL_CO_) and square root‐transformed expression of *INPP4A*. GraphPad Prism (version 8) software was used for the statistical analysis.

## 3 RESULTS

### 3.1 INPP4A transcription is elevated in IPF

INPP4A is universally expressed in all human tissues with maximum expression in brain, lymph node and spleen as per the National Centre of Biotechnology Information (NCBI) database (Figure S1a, https://www.ncbi.nlm.nih.gov/gene/3631). RNA sequencing data of normal subjects showed that INPP4A expression was similar across different age groups and was relatively expressed more in immune cells in adult lung as per the lungMAP database [Figure S1b (www.lungmap.net; Ardini‐Poleske et al. (2017))]. For additional insights into different cell types expressing INPP4A in IPF lungs, single cell RNA sequencing data of IPF patients from GSE136831 dataset was used. Figure S1c shows the INPP4A expression across different cell types in IPF lungs. INPP4A was expressed in a wide variety of cell types and immune cells showed relatively higher expression compared to epithelial, mesenchymal and endothelial cell types [Figure S1c (Adams et al., 2020)]. These results show that INPPA is expressed in many diverse cell types both in normal and IPF lungs.

**FIGURE 1:**
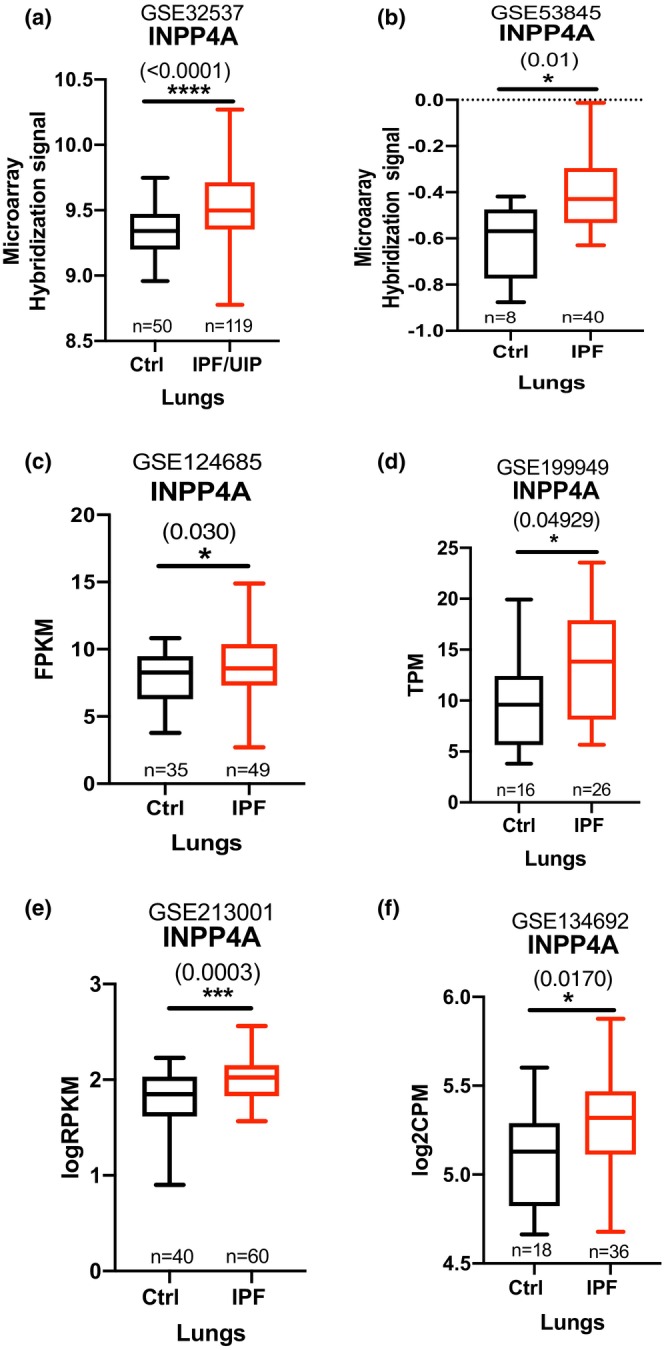
Inositol Polyphosphate‐4‐Phosphatase Type I A (*INPP4A*) is upregulated in Idiopathic Pulmonary Fibrosis. Normalized *INPP4A* gene expression data from control and IPF whole lungs in microarray based GEO datasets namely (a) GSE32537 and (b) GSE53845 and RNA‐sequencing based GEO datasets namely (c) GSE124685 (d) GSE199949 and (e) GSE213001 and (f) GSE134692 (only batch 1 samples were considered for normalizing batch effects). No of subjects in each category is indicated on all graphs as “*n*.” Information about datasets also provided in Table 2. Statistical significance was calculated using unpaired *t*‐test for (d) and (f), unpaired *t*‐test with Welch's correction in (a) and (c), Mann–Whitney test for (b) and (e). Relative expression levels are represented as fragments per kilobase of transcript per million mapped reads (FPKM) or reads per kilobase of exon per million reads mapped (RPKM) or transcript per million (TPM) or counts per million (CPM). The numbers in parentheses are indicative of *p*‐values for statistical significance. *p < 0.05, **p < 0.01, ***p < 0.001, ****p < 0.0001. Ctrl, Control; IPF, idiopathic pulmonary fibrosis; UIP, usual interstitial pneumonia.

INPP4A, a modulator of PI3K‐AKT pathway, is known for its role in the regulation of fundamental processes of the cell such as cell proliferation and apoptosis. Previously, we have demonstrated the implications of INPP4A downregulation, especially the secretory form, in the regulation of airway inflammation and sub‐epithelial fibrosis during asthma (Khanna et al., 2019). To understand if this was relevant to other fibrotic lung diseases, particularly IPF, we studied INPP4A expression in pulmonary fibrosis. For this purpose, we used publicly available microarray and RNA‐sequencing datasets relevant to lung fibrosis. We extracted processed data from 6 datasets from the GEO database (Table 2) (DePianto et al., 2015; Huang et al., 2022; Jaffar et al., 2022; McDonough et al., 2019; Sivakumar et al., 2019; Yang et al., 2013). Our analysis revealed that *INPP4A* is upregulated in human lung samples of idiopathic pulmonary fibrosis, compared to control lung samples. This observation was consistent across different microarray and RNA‐sequencing datasets analyzed (Figure 1a–f).

### 3.2 INPP4A gene expression in lung is associated with clinically relevant differences in patients with IPF

**FIGURE 2:**
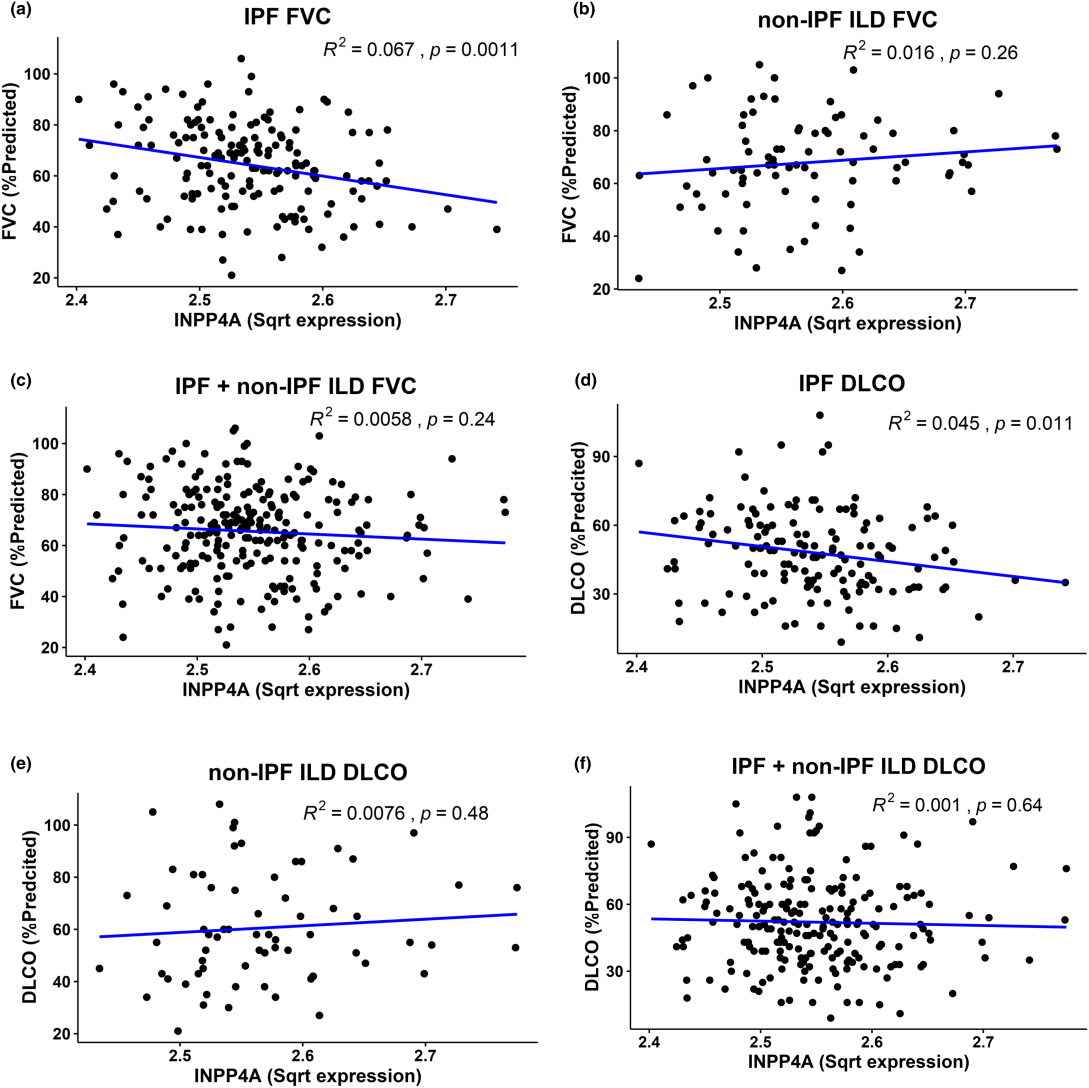
Inositol Polyphosphate‐4‐Phosphatase Type I A (*INPP4A*) gene expression correlates with forced vital capacity (FVC) and diffusing capacity of the lung for carbon monoxide (DLCO) in idiopathic pulmonary fibrosis. Pearson correlation coefficients of FVC as a function of lung tissue expression of INPP4A for (a) IPF; *n* = 157 (b) non‐IPF interstitial lung disease (non‐IPF ILD); *n* = 82 (c) combined cohort of IPF and non‐IPF ILD patients; *n* = 239. Pearson correlation coefficients of DL_CO_ as a function of *INPP4A* gene expression in lung tissues for (d) IPF; *n* = 145 (e) non‐IPF ILD; *n* = 67 and (f) combined cohort of IPF and non‐IPF ILD; *n* = 212. Data for lung function parameters, percent predicted FVC and percent predicted DL_CO_ and *INPP4A* lung tissue expression were extracted from lung tissue research consortium (LTRC) dataset GSE47460. *INPP4A* square root (sqrt) expression denotes sqrt–transformed hybridisation signal intensities from the mentioned microarray dataset. *R* ^2^ (where *R* values denote Pearson correlation coefficient) and *p*‐values are mentioned in the figure. *n* indicate number of subjects/samples in each case. ILD, interstitial lung disease; IPF, idiopathic pulmonary fibrosis.

To understand the relevance of the observed increase in *INPP4A* to IPF disease pathophysiology, we quantified the association of *INPP4A* expression with key physiological parameters. Changes in forced vital capacity (FVC) and diffusion capacity of lung for carbon monoxide (DL_CO_) have often been used to define IPF improvement, stability or progression. Here we quantified the association between the INPP4A expression and lung function indicators FVC or DL_CO_ using cross‐sectional data available from Lung Genomics Research consortium (GSE47460). Using univariate regression analysis, we observed that *INPP4A* gene expression in lung tissues predicted FVC in IPF. Increased *INPP4A* expression was significantly associated with decrease in lung function indicated by low % predicted FVC in IPF samples (Figure 2a). Of note, this association was not observed in case of non‐IPF ILD cohort (Figure 2b) and lost when measured for a combined set of samples containing both IPF and non‐IPF ILD (Figure 2c) indicating the specificity of this association in IPF cohort. We repeated the same analysis with % predicted DL_CO_ measurements available in the same dataset. Similar to our observations in case of FVC, we found that *INPP4A* whole lung levels also predicted DLCO in IPF patients (Figure 2d). This was not observed in non‐IPF ILD (Figure 2e) or the combined cohort of IPF and non‐IPF ILD patients (Figure 2f). Thus, IPF patients may be distinguished clinically based on INPP4A gene expression.

### 3.3 INPP4A protein levels are elevated in IPF and bleomycin induced pulmonary fibrosis

**FIGURE 3:**
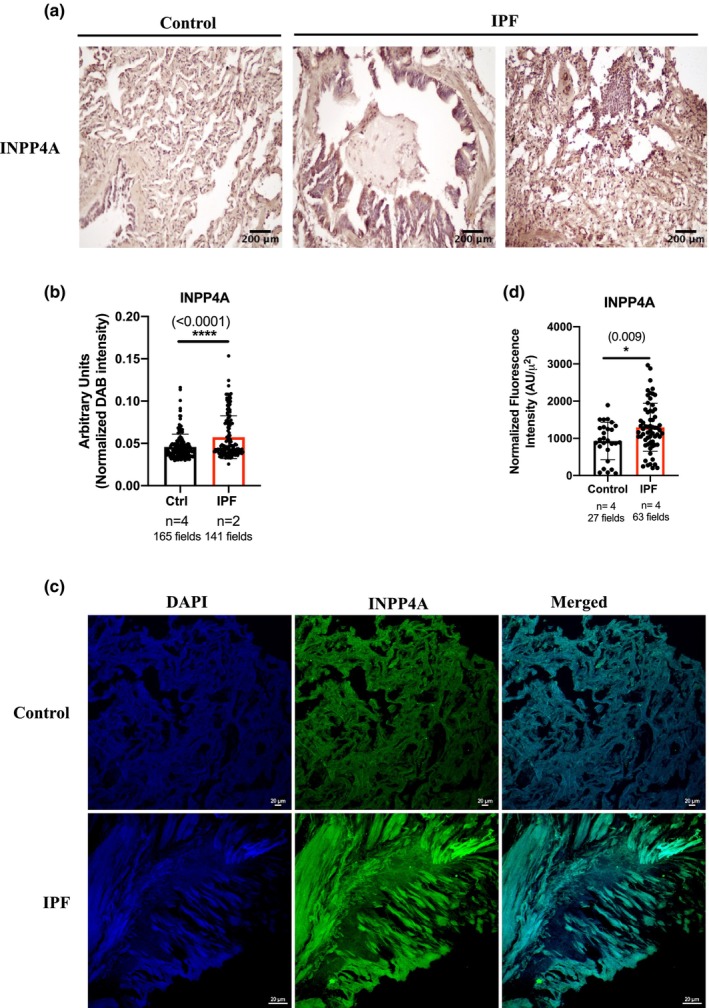
Inositol Polyphosphate‐4‐Phosphatase Type I A (INPP4A) expression is increased in pulmonary fibrosis. (a) Representative images for immunohistochemical staining performed for INPP4A on lung tissue sections of control and IPF patients. Bronchiolar and alveolar regions of IPF lung tissues were labeled to show INPP4A expression in these regions. Protein of interest can be seen brown colored due to DAB staining and nuclei are labeled blue due to hematoxylin staining. Scale bars indicate 200 μm. Original magnification is 20×. (b) Quantification of Figure 3a. Normalized DAB intensity indicates normalization of total protein expression value by value of number of nuclei in each section/image. Number of fields unbiased and random, for covering the entire lung section with high confidence are mentioned in the figure. (c) Representative immunofluorescence confocal microscopy images used to study INPP4A expression. INPP4A (green), nuclei (blue). Scale bar = 20 μm. Original magnification 20× (d) INPP4A quantification from Figure 3c. Number of fields per sample are indicated. *n* indicates number of subjects/samples in each case. Statistical significance was calculated using Mann–Whitney test for (b) and unpaired *t*‐test for (d). The numbers in parentheses are indicative of *p*‐values for statistical significance. *p < 0.05, **p < 0.01, ***p < 0.001, ****p < 0.0001. IPF, idiopathic pulmonary fibrosis.

To confirm whether changes in *INPP4*A expression reflect changes in lung fibrosis at the protein level, INPP4A positivity was examined using immunohistochemistry in formalin fixed paraffin‐embedded sections obtained from IPF patients and non‐diseased control donors. Strong INPP4A expression was observed in both the control and diseased human lungs. Furthermore, INPP4A expression was increased in IPF patient lungs compared to control lungs (Figure 3a). We used the integrated semiquantitative IHC scoring protocol published by Crowe and Yue in 2019 (Crowe & Yue, 2019) as explained in methods and performed quantification of INPP4A^+^ cells in the INPP4A immune‐stained sections using the same (Figure 3b) which confirmed increased INPP4A expression in IPF. Fluorescence based immunostaining of INPP4A was also performed in the lung sections of same subjects, further validating the upregulation of INPP4A at the protein level in IPF lungs (Figure 3c, quantified in Figure 3d).

**FIGURE 4:**
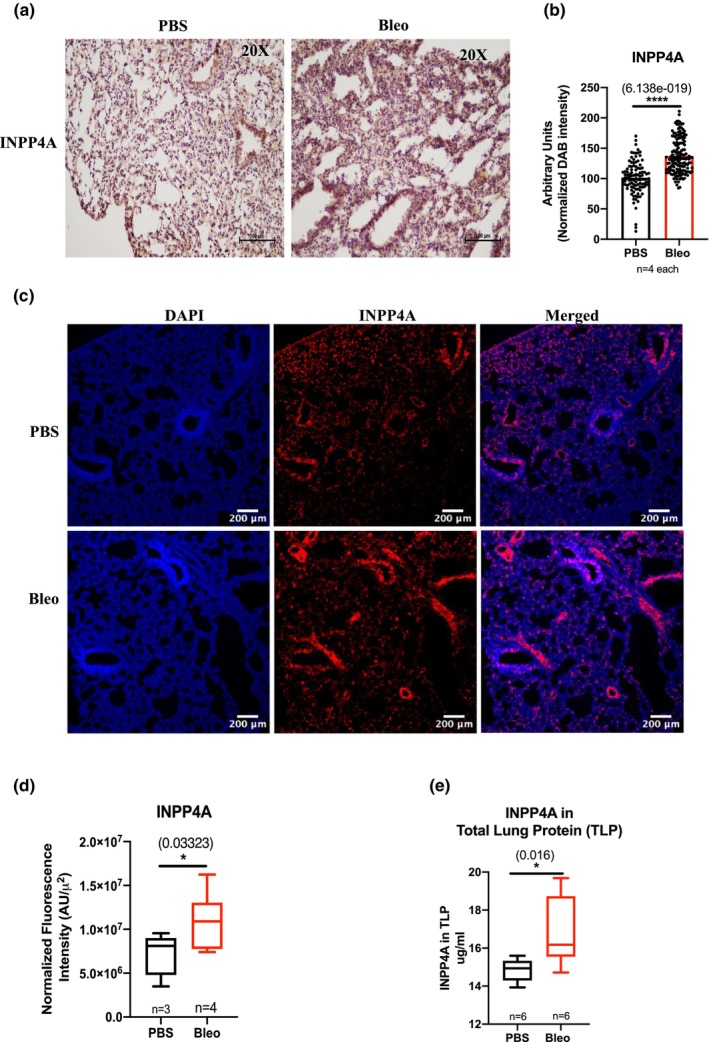
Inositol Polyphosphate‐4‐Phosphatase Type I A (INPP4A) expression is increased in bleomycin induced pulmonary fibrosis in mice. (a) Representative images for immunohistochemical staining performed on lung tissue sections to determine INPP4A expression in vehicle (PBS) and bleomycin (Bleo) treated mice. Interstitial or alveolar lung regions shown here. Protein of interest appeared brown due to DAB staining and nuclei were labeled blue due to hematoxylin staining. Scale bars indicate 100 μm. Original magnification is 20×. (b) Quantification of Figure 4a. Normalized DAB intensity indicates normalization of total protein by number of nuclei in the field. Number of fields imaged per sample are indicated in the figure. DAB: 3,3′‐Diaminobenzidine. (c) Representative immunofluorescence confocal microscopy images of vehicle/PBS (*n* = 3) and bleomycin treated mice (*n* = 4) lung tissue sections stained with anti‐INPP4A (red) and nuclear stain, DAPI (blue). Original magnification is 20× (d) Quantification of Figure 4c (e) Cellular or tissue level INPP4A expression in total lung protein (TLP) of vehicle and bleomycin treated mice, determined by ELISA. Statistical significance was calculated using Mann–Whitney test in (b) and (d) and using unpaired *t*‐test in (d). The numbers in parentheses are indicative of *p*‐values for statistical significance. *p < 0.05, **p < 0.01, ***p < 0.001, ****p < 0.0001.

With the understanding that INPP4A expression is relevant in IPF pathophysiology, we sought to investigate the therapeutic potential of experimental downregulation of INPP4A in pulmonary fibrosis. We first determined whether bleomycin‐induced lung injury mimics changes in INPP4A expression observed in human IPF. For generating this acute model of pulmonary fibrosis, C57BL/6 mice were administered with a single dose of bleomycin (Bleo) intratracheally on Day 0. The mice were then sacrificed on Day 21 post bleomycin treatment. Lung function and extent of inflammation and fibrosis were assessed to confirm establishment of the model (Figure S2a–e). To determine whether increased inflammation, collagen deposition and architectural distortion in bleomycin‐treated mice lungs were accompanied by an altered expression of INPP4A, we performed immunohistochemical staining of mice lungs treated with vehicle and bleomycin for INPP4A. INPP4A positive cells were abundant at baseline, in PBS treated mouse lungs but increased significantly following bleomycin injury as observed in the interstitial region of mice lungs (Figure 4a, quantified in Figure 4b). Greater INPP4A expression was consistent with the findings of increased fibrosis in lung sections of Bleo mice noted in Figure S2. We also examined INPP4A expression in mice lung tissues by immunofluorescence to validate our histological observations. In the lung tissues from PBS‐treated mice, signals of INPP4A were only sparsely observed. By contrast, INPP4A signals were clearly observed in bleomycin‐treated lung tissues (Figure 4c), further validated by significantly increased levels after quantification (Figure 4d).

To further confirm upregulated INPP4A protein expression in Bleo mice, we performed ELISA on lung homogenates, derived from mice with bleomycin induced pulmonary fibrosis and vehicle treated mice. We detected a very small but significant increase in the levels of INPP4A protein in Bleo lungs compared to control (Figure 4e).

### 3.4 INPP4A is heterogeneously expressed in pulmonary fibrosis with higher expression in lesser fibrotic regions than highly fibrotic ones

**FIGURE 5:**
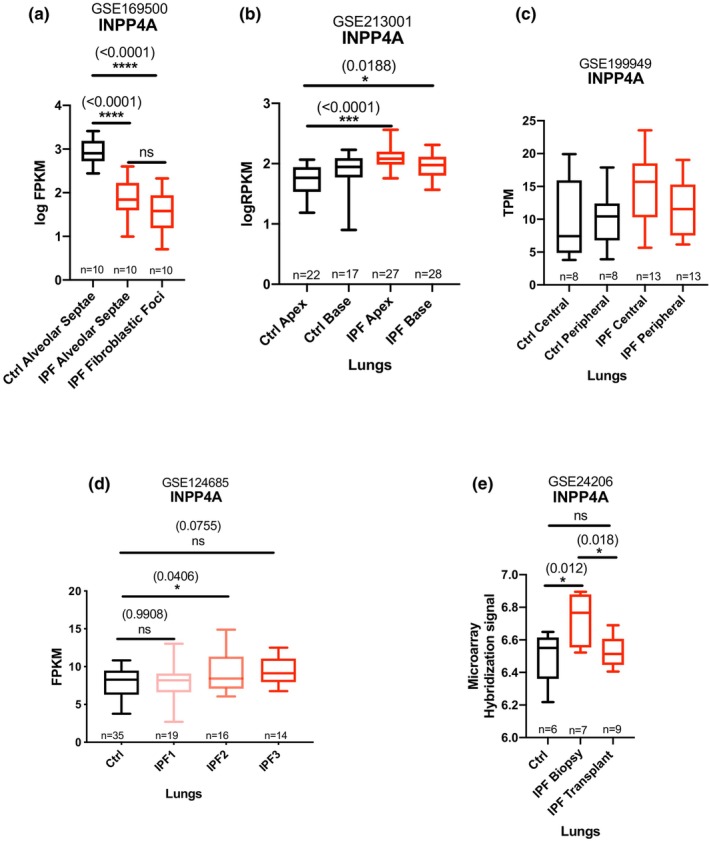
*INPP4A* gene dysregulation is highest in non‐fibrotic or lesser fibrotic IPF tissues. Box plot representing median and interquartile ranges using data mined from RNA sequencing based datasets for (a) *INPP4A* expression in control alveolar septae, IPF alveolar septae and IPF fibroblastic foci isolated using laser microdissection in GEO dataset GSE169500. (b) *INPP4A* gene expression levels in selected lung tissue regions of non‐disease control donors and IPF patients; in GEO dataset GSE213001. Base and apex denote samples derived from base (end‐stage) and matched lung apices (pre‐terminal disease) lung regions from controls and IPF patients. (c) *INPP4A* expression in GEO dataset GSE199949. Sample labelling indicates macroscopically non‐fibrotic central (IPF central) and fibrotic peripheral (IPF peripheral) areas. Donor lung, non‐IPF biopsies are labeled similarly as central (Ctrl central) and peripheral (Ctrl peripheral). (d) *INPP4A* expression in isolated lung lobes of IPF categorized as control/early‐stage IPF (IPF1), progressive IPF2 (IPF2), and severe/end‐stage IPF (IPF3) based on micro CT and Ashcroft staining (histological staining) from GEO dataset GSE124685 (e) Differential expression of *INPP4A* in control and IPF lung biopsy and transplant samples in microarray dataset GSE24206. Additional information about datasets used here in Table 3. Statistical significance was calculated using one‐way ANOVA with Tukey's multiple comparison's test for (a), Kruskal–Wallis test for (b), unpaired *t*‐test for (c), one way ANOVA with Dunnett's multiple comparison test for (d) and one way ANOVA for (e). RPKM: reads per kilobase million; TPM: Transcript per million. FPKM: Fragments per Kilobase of transcript per Million mapped reads. The numbers in parentheses are indicative of p values for statistical significance between the marked groups. *p < 0.05, **p < 0.01, ***p < 0.001, ****p < 0.0001, ns, non‐significant. *n* indicates no of subjects in each case.

In the above section, INPP4A expression was found to be upregulated in whole lung samples of pulmonary fibrosis. Since IPF is characterized by usual interstitial pneumonia (UIP), which has hallmarks of spatial heterogeneity that is, the occurrence of fibrotic lung near histologically normal lung and temporal heterogeneity that is, the acutely active disease present along with progressively active disease marked by fibrotic scars, we further wished to understand if INPP4A expression was uniform across heterogeneous regions of IPF (Raghu et al., 2018, 2022). Therefore, we looked into finer resolution, that is, the fibroblastic foci (FF), which are regions of active fibrogenesis in IPF (Katzenstein & Myers, 1998) and hypothesized that the increase in INPP4A expression in IPF lungs must be reflected by the increase in INPP4A expression in the pathological core of pulmonary fibrosis, the fibroblastic foci. To determine the same, we reanalyzed different datasets generated to understand the gene expression changes associated with spatiotemporal heterogeneity observed in human IPF and mouse models of fibrosis. The details of the datasets used are presented in Table 3. First, we reanalyzed a dataset containing transcriptome profiling of discrete regions of fibroblastic foci and alveolar septae extracted from fixed lung sections of IPF and healthy subjects (GSE169500) (Brereton et al., 2022). To our surprise, *INPP4A* expression was observed to be significantly reduced in the fibroblastic foci region of IPF lungs compared to control alveolar septae (Figure 5a) and was in contradiction to that of patterns of INPP4A expression in the bulk PF tissues. Moreover, in the same dataset, *INPP4A* expression levels showed moderate to strong correlation with expression of various well established pro‐fibrotic marker genes such as *MUC5B*, *ACTA2*, *COL1A1*, *EPCAM*, and *FN1* (Figure S3) that were differentially expressed between IPF fibroblastic foci and control alveolar septae (Figure S4).

Given the observed heterogeneous expression of INPP4A in IPF from the data above, we were curious to profile other regions of lungs with PF for INPP4A expression. For this, studies that performed transcriptomic profiling of temporally and spatially different regions of fibrotic tissues varying in their degree of fibrosis, were investigated. In GSE213001, whole transcriptome of bulk apex and base was sequenced for tissues derived from IPF patients where IPF lung tissue bases (end stage) were found to be more fibrotic than the apices (pre‐terminal disease) by the authors (Jaffar et al., 2022). Interestingly, in the above dataset, *INPP4A* levels were elevated in the less fibrotic, IPF apex tissues compared to control apex lung tissues. Further, *INPP4A* expression increased in IPF base tissues only when compared to control apex lung tissues and not when compared to control base ones (Figure 5b). Also, difference in *INPP4A* levels were not observed between IPF apices and bases or between control apices and bases. A similar trend could be seen in the data plotted from GSE199949 where RNA sequencing was carried out in paired biopsies from peripheral (fibrotic) and central (non‐fibrotic) lung regions in a cohort of non IPF donors and IPF patients (Huang et al., 2022), however no significant difference was observed (Figure 5c). We also reanalyzed another RNA sequencing dataset (GSE124685) where the authors tried to capture and categorize spatial and temporal heterogeneity by sequencing different regions of a fibrotic lung (McDonough et al., 2019). McDonough et al, in this report, divided each IPF lung into three parts and lobes were isolated from each part. Further these lobes were classified into IPF1, IPF2 and IPF3 representing low, moderate and severe fibrosis respectively, based on micro CT and Ashcroft scores. We wished to see how INPP4A expression varied in these spatial regions of IPF tissues, varying in the degree of fibrosis. We observed that compared to control, differential expression of *INPP4A* was not present in regions of the lung that looked normal during histological assessment (IPF1). Rather gene expression changes in *INPP4A* were seen to occur only in histologically mildly fibrotic regions such as IPF2 and again appeared insignificant in severely fibrotic tissue regions (IPF3) (Figure 5d). Another dataset (GSE24206) depicted that relative to control, *INPP4A* levels were significantly high in biopsy/moderate stage IPF samples but not in transplant/advanced IPF samples (Figure 5e) (Meltzer et al., 2011). Here, significant differential gene expression of *INPP4A* was also observed between IPF biopsy and IPF transplant lung tissue samples.

**FIGURE 6:**
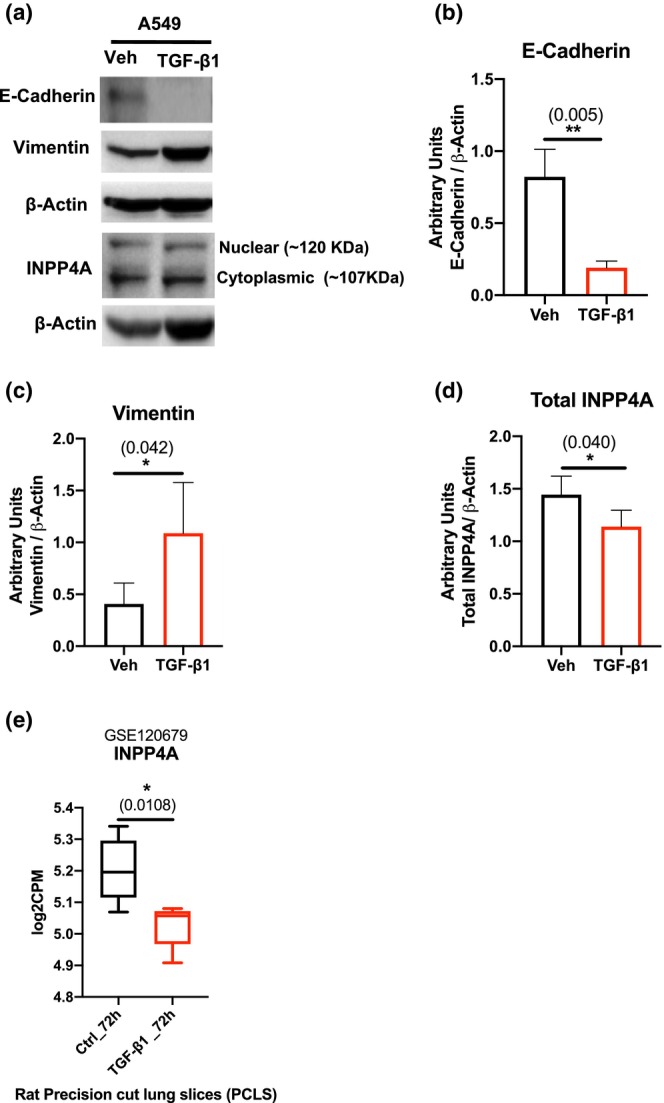
INPP4A Expression is reduced in TGF‐β1 induced myofibroblasts. (a) Western blot analysis of alveolar epithelial cells (A549) treated with vehicle (Veh) or TGF‐β1 for 120 h.; total cell lysates were extracted and analyzed for expression of E‐cadherin, vimentin and INPP4A using immunoblotting. β‐Actin was used as an internal control. Densitometric quantification of Figure 6a for (b) E‐Cadherin and (c) Vimentin and (d) Total INPP4A (sum of cytoplasmic INPP4A that is, 107KDa and nuclear INPP4A that is, 120KDa band intensities). (e) *INPP4A* levels in TGF‐β1 treated rat precision cut lung slices (PCLS), GSE120679. Relative expression levels are represented as counts per million (CPM). The numbers in parentheses are p values indicating statistical significance calculated using unpaired *t*‐test in each graph. *p < 0.05, **p < 0.01, ***p < 0.001, ****p < 0.0001. Data shown are means ± SD. Minimum number of independent experiments in each case is three.

Since INPP4A expression was significantly altered in human and mice samples relevant to IPF, we further wished to check its expression in in vitro models relevant to IPF pathophysiology. Development of idiopathic pulmonary fibrosis depends on the conversion of lung fibroblasts into smooth muscle actin (α‐SMA)‐positive myofibroblasts which are the primary constituents of fibroblastic foci. Transforming growth factor‐β (TGF‐β) is a master profibrotic cytokine that is extremely important in the development of lung fibrosis and TGF‐β mediated EMT of alveolar epithelial cells (AECs) has been suggested to have prominent roles in the formation of myofibroblasts and progression of pulmonary fibrosis (Frangogiannis, 2020). Therefore, we performed INPP4A immunoblotting after TGF‐β1 mediated EMT induction in an AECII line, A549. Since the largest difference in morphological and molecular EMT related changes between the control and treatment groups was seen 120 h after stimulation (data shown only for 120 h timepoint, Figure S5; Figure 6a–c), we further studied INPP4A levels at this timepoint post TGF‐β1 treatment. The results show that TGF‐β1 induced EMT as observed by decreased E‐cadherin levels (Figure 6a,b) and increased vimentin levels (Figure 6a,c), was accompanied by suppressed total INPP4A (sum of cytoplasmic and nuclear INPP4A) expression (Figure 6a,d). We also investigated the effects of TGF‐β1 on *INPP4A* expression in a rat precision cut lung tissue slice (PCLS) model using GSE120679 (Huang et al., 2019). Comprising of all major lung cell types and preserving the natural cell–cell contacts and cell matrix contacts, PCLS serves as a promising ex‐vivo model system for studying pulmonary fibrosis. We saw reduced transcription of INPP4A to be amongst the robust fibrotic responses seen post TGF‐β1 induction (GSE120679, Figure 6e).

Additionally, reanalysis of two other publicly available datasets confirmed similar trends of expression where in one, *INPP4A* was found to have reduced expression in myofibroblasts from bleomycin‐treated mice lungs when compared with steady‐state fibroblasts from untreated lungs (GSE111043, Figure S6a) (Enomoto et al., 2018). Fibroblasts from bleomycin injured aged mice also showed reduced *INPP4A* expression in comparison to fibroblasts from bleomycin injured young mice indicating attenuated *INPP4A* expression to be a part of transcriptional programming governing persistent lung fibrosis (GSE191208, Figure S6b) (Pham et al., 2022).

### 3.5 Anti‐INPP4A antibody worsens fibrosis in bleomycin induced PF model

**FIGURE 7:**
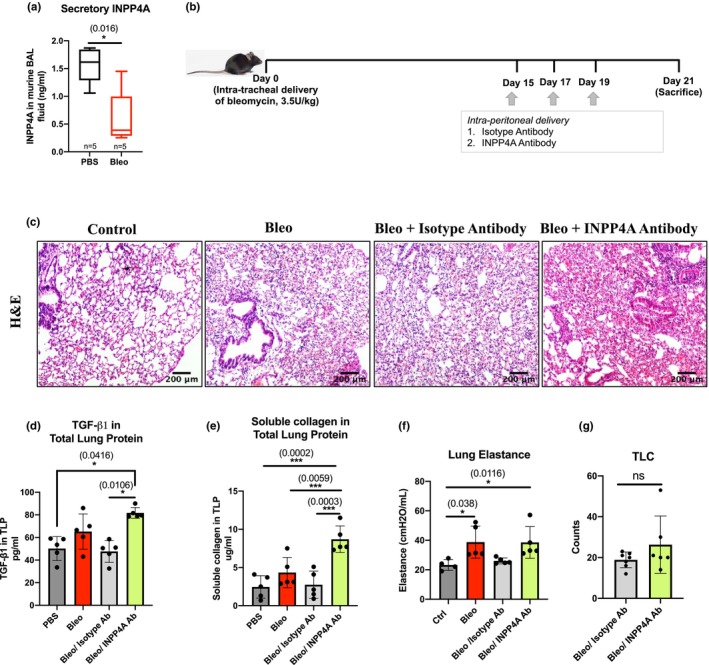
Secretory INPP4A is protective in mice with bleomycin induced pulmonary fibrosis. (a) Measurement of secretory/extracellular INPP4A levels in bleomycin treated mice by measuring INPP4A expression in bronchoalveolar lavage (BAL) fluid determined by ELISA. *n* represents number of mice in each case. (b) Schematic of experimental protocol used for anti‐INPP4A antibody treatment in bleomycin treated mice with pulmonary fibrosis (c) Representative images for H&E stained sections for different groups as mentioned. Original magnification is 10×. Scale bars represent 200 μm (d) TGF‐ β1 cytokine levels in TLP prepared from lungs of mice treated with isotype or anti‐ INPP4A antibody as measured by ELISA (e) Amount of soluble collagen in the lungs of mice treated with isotype or anti‐INPP4A antibody measured by Sircol assay (f) Lung elastance measurement after treatment with anti‐INPP4A or isotype antibody in mice induced with bleomycin (g) Total leukocyte count (TLC) in mice given bleomycin followed by anti‐INPP4A antibody treatment. TLP = total lung protein. Statistical significance was calculated using Mann–Whitney test for (a), Kruskal–Wallis test for (c) and (f), one way ANOVA with Tukey's multiple comparisons test for (d) and Mann–Whitney test for (e). The numbers in parentheses are indicative of respective p values as marked between the groups. *p < 0.05, **p < 0.01, ***p < 0.001, ****p < 0.0001. ns, not significant. Number of animals is five or more in each group as indicated by dots. Data shown are means ± SD.

INPP4A's heterogeneous expression as discussed in the previous section opened up two possibilities of its role in pulmonary fibrosis, compensatory or pathogenic. To definitively confirm that, we treated bleomycin induced mice using an intraperitoneally injected anti‐INPP4A antibody. Secretory INPP4A (extracellular form) is known to play a key role in the regulation of airway inflammation and sub‐epithelial fibrosis (Khanna et al., 2019). Before assessing the effect of INPP4A neutralization, we first checked if INPP4A secretion was altered in pulmonary fibrosis. To decipher the same, we checked the levels of secretory INPP4A in BALF of mice treated with bleomycin. In contrast to the increased cellular INPP4A levels in IPF lung tissues, secretory INPP4A levels were reduced in BALF from Bleo mice (Figure 7a).

We next proceeded to distinguish between the compensatory and pathogenic roles of INPP4A in pulmonary fibrosis. To explore this, antibody treatment was given to mice with bleomycin induced pulmonary fibrosis. We administered anti‐INPP4A antibody or the control isotype antibody thrice during the fibrotic phase (starting at Day 15 post bleomycin) to mice treated with bleomycin on Day 0 (Figure 7b). Subsequently we assessed pathology, fibrosis and lung function at Day 21 post bleomycin. Mice with bleomycin induced PF given anti‐INPP4A antibody had worsened pulmonary fibrosis in comparison to their respective controls (Figure 7c–e). Increased inflammation and cellular infiltration (Figure 7c) were accompanied by concomitant increase in levels of the primary pro‐fibrotic cytokine TGF‐β1 in the lung tissue lysates of these mice (Figure 7d). The amount of soluble collagen was also increased significantly upon treatment with anti‐INPP4A antibody in Bleo mice (Figure 7e). However, these pro‐fibrotic changes weren't accompanied by lung function alteration after administration of anti‐INPP4A antibody to Bleo mice in comparison to isotype antibody control groups, as measured by respiratory elastance (Figure 7f). BAL total cell counts also remained unaltered in Bleo mice after anti‐INPP4A Ab administration (Figure 7g). Taken together the above data suggest that anti‐INPP4A antibody treatment aggravates bleomycin induced pulmonary fibrosis in mice with no or limited effect on lung elastance. These results suggest an important role for INPP4A compartmentalization, specifically secretory INPP4A in pulmonary fibrosis and warrants further investigation.

## 4 DISCUSSION

IPF is a devastating disease with poor prognosis and survival post diagnosis. Progressive fibrosis of lung interstitium, as a result of activation and hyperproliferation of fibroblasts, is a key pathological feature of IPF. Though there are several reports on the INPP4A mediated regulation of fibroblast survival and proliferation (Ivetac et al., 2009; Khanna et al., 2019; Vyas et al., 2000), its regulation in IPF is underexplored. Here, we present the collated results from relevant publicly available microarray and RNA‐seq datasets, mouse models, and in vitro experiments on lung epithelial cells, to provide insights into the differential regulation of INPP4A during IPF. Together, our findings show that INPP4A is significantly altered in IPF, but the direction of alteration is dependent on cellular state, extent of fibrosis in the sampled lung region and the disease stage at which it is being measured.

INPP4A belongs to the family of lipid phosphatases involved in the maintenance of cellular levels of phosphoinositide species. This enzyme has an N‐terminal C2 domain for lipid binding, a PEST domain for protein–protein interactions and a C‐terminal domain containing C(X)5R active motif which determines its phosphatase activity (Ivetac et al., 2005). Knock‐out studies have revealed that INPP4A plays a critical role in protecting neurons from glutamate induced excitotoxicity, and its loss results in severe neurological phenotype and death within 2–3 weeks after birth (Sasaki et al., 2010). In line with this, INPP4A is highly expressed in brain. Other than brain, it is also expressed in different cell types of various tissues including lungs (Figure S1a). Apart from neuroprotection, INPP4A has been shown to regulate cell proliferation and survival in a number of cell types including fibroblast cells (Chaudhuri et al., 2018; Ivetac et al., 2009; Khanna et al., 2019; Morioka et al., 2016; Vyas et al., 2000), and has been implicated in cancer (Chaudhuri et al., 2018; Erkeland et al., 2004; Huang et al., 2019; Jiang et al., 2015; Lin et al., 2014; Maher et al., 2009; Mäki‐Nevala et al., 2016; Thutkawkorapin et al., 2019; Wang et al., 2018). Previous reports from our lab have demonstrated a crucial role for INPP4A in asthma and allergic airway inflammation (Aich, Mabalirajan, Ahmad, Khanna, et al., 2012; Khanna et al., 2019; Sharma et al., 2008). Additionally, secretory INPP4A has been shown to be protective in peri‐bronchial fibrosis and unrestricted fibroblast proliferation in vitro (Khanna et al., 2019). Given the relevance of INPP4A in the survival and proliferation of fibroblast cells and in lung homeostasis, we hypothesized that INPP4A may play a role in IPF. To test this hypothesis, we analyzed relevant publicly available human transcriptional data from GSE32537, GSE53845, GSE124685, GSE199949, GSE213001, and GSE134692 datasets for differential expression of *INPP4A*. We found that there is significant increase in *INPP4A* gene expression in the lungs of IPF patients. The specificity of *INPP4A* upregulation observed in these datasets was supported by similar findings in an independent set of lung samples from IPF and control donors collected at Mayo clinic, Rochester, USA, and from studies on bleomycin‐induced mouse models of pulmonary fibrosis (Figures 3 and 4). Also, univariate analysis of the cross sectional LTRC dataset (GSE47460) showed a negative association between *INPP4A* expression and lung function measures, % predicted FVC and DL_CO_. This association was specifically observed in IPF patients, but not in non‐IPF ILD patients suggesting that *INPP4A* upregulation is clinically relevant. However, the causal nature of this finding requires further validation. Though these results suggest upregulation of INPP4A in IPF, caution is required while interpreting such data citing the heterogeneity in IPF. It is important to note that the presented data belongs to bulk transcriptomics studies and thus represents the average *INPP4A* expression of multiple cell types present in the lung which may dynamically vary depending on the lung region and disease stage at which the samples were collected. Recent transcriptomic studies have highlighted how gene expression profiles vary with the heterogeneity of IPF disease (Jaffar et al., 2022; McDonough et al., 2019; Meltzer et al., 2011). Therefore, it is important to verify the effects of disease heterogeneity on the INPP4A expression.

To understand whether the changes in INPP4A expression were independent of disease stage and regional heterogeneity observed in IPF lungs, we analyzed publicly available GSE169500, GSE213001, GSE199949, GSE124685, and GSE24206 datasets. All these datasets, except GSE169500 and GSE24206, were originally generated to investigate the gene expression changes through the progression of IPF by exploiting the regional heterogeneity observed in the disease. On the other hand, GSE169500 was generated to understand the differences in the gene expression of alveolar septa and fibrotic foci from lung samples of IPF patients compared to alveolar epithelium of non‐disease control lungs. Similarly, GSE24206 was generated with an intent to develop gene expression signature that could discriminate IPF samples from healthy subjects. Analysis of these datasets revealed that *INPP4A* was significantly upregulated only in the less fibrotic that is, apex [GSE213001] regions of the lung, but not in the more fibrotic that is, base [GSE213001] regions (Figure 5b). In another dataset, GSE199949, INPP4A expression was high in central lung regions of IPF samples compared to the similar regions in control samples, but the results were not statistically significant (Figure 5c). Similar findings were made in GSE124685 dataset which contained transcriptomic data of lung tissue samples that were classified as early, progressive or end‐stage fibrosis based on micro‐CT quantitative imaging and histology. Further, analysis of data from GSE24206 showed that *INPP4A* levels were high in early‐stage biopsy samples from IPF patients compared to healthy donors and explant samples collected from IPF patients during lung transplantation. Together, these results suggest that INPP4A expression follows a non‐linear pattern through the disease course, and the increased expression observed in bulk IPF lungs might be a reflection of INPP4A expression in lesser fibrotic regions or seemingly unaffected areas of IPF tissues than the severe fibrotic regions such as fibroblastic foci. If this is the case, the individual cell types contributing to the disease should reflect these changes. Indeed, we found that *INPP4A* is downregulated in myofibroblasts isolated from young and aged mice treated with bleomycin (GSE111043 and GSE191028; Figure S6) compared to controls, and in alveolar septae bounding the fibroblastic foci (GSE169500; Figure S5a). Similar findings were observed in rat precision‐cut lung slices, and A549 cells treated with TGF‐β1 (Figure 6). Additionally, pirfenidone treatment was found to revert *INPP4A* levels in rat precision‐cut lung slices treated with TGF‐β1 (Figure S7a; GSE120679) (Huang et al., 2019). Similarly, in a different study, nintedanib was found to improve *INPP4A* levels in fibroblasts derived from IPF lung samples (Figure S7b) (Sheu et al., 2019). Weak to moderate, but significant, correlation was observed between INPP4A and pro‐fibrotic, and epithelial genes suggesting a possible co‐regulation of these genes downstream to TGF‐β1. These results along with increased expression of some of the pro‐fibrotic genes in alveolar septae from IPF patients suggest that downregulation of INPP4A may be associated with epithelial mesenchymal transition (EMT) reported in IPF (Figure S3). This leads us to the question of how INPP4A might contribute to the EMT process. INPP4A has been shown to negatively regulate cell proliferation and apoptosis of many cell types including fibroblasts (Chaudhuri et al., 2018; Ivetac et al., 2009; Vyas et al., 2000; Wang et al., 2018). Therefore, we speculate that loss of INPP4A might be playing a role in reducing alveolar epithelial cell apoptosis and thus promoting their transformation to fibroblasts through EMT. Additionally, loss of INPP4A may also promote aberrant fibroblast hyperproliferation and thus establishment of fibrosis. Together, these results suggest that the observed lack of difference in the INPP4A expression, relative to healthy lungs, during advanced stages of IPF could be due to heterogeneity in the extent of fibrosis in the sampled lung regions. Though our results suggest that the INPP4A upregulation observed at the whole tissue level is largely due to over representation of less fibrotic tissue in the sampled lung region, they do not explain the cell types contributing to the observed increase in INPP4A levels in less fibrotic regions. In a previous report, Nuovo et al., (Nuovo et al., 2012) have shown that fibrotic lung regions of IPF patients contain fewer immune cells. Therefore, we assume that changes in the immune cell composition of the lung may explain the INPP4A upregulation during early disease. This claim could be partially supported by the fact that immune cells have the second highest levels of INPP4A (Figure S1b). Also, single cell transcriptomics data from IPF lung samples showed that immune cells have higher cellular expression compared to other cell types (Figure S1c). While this might explain the upregulation of INPP4A, its contribution to the IPF disease pathogenesis remains unclear. Also, considering the heterogeneity in the INPP4A expression it is possible that the observed changes in the INPP4A could be a compensatory response with no relevance to the IPF pathogenesis.

We have previously shown that INPP4A is secreted by bronchial epithelial cells into the extracellular environment where it regulates the sub‐epithelial fibroblast proliferation during allergic airway inflammation (Khanna et al., 2019). In line with this, we found that INPP4A secretion was reduced in the BAL fluid of bleomycin‐treated mice. Its pathological relevance was supported by exaggeration of fibrosis by intraperitoneal anti‐INPP4A antibody treatment in bleomycin‐treated mice. Lung histology and increased expression of pro‐fibrotic markers in response to anti‐INPP4A antibody treatment support these findings (Figure 7). Absence of any further increase in the lung elastance post‐INPP4A antibody treatment may be due to limited time between antibody treatment and sacrifice.

Although, based on the current results it is difficult to ascertain the role of INPP4A in the pathophysiology of IPF, one way to reconcile our results is that fibrotic areas of the lung have burnt out inflammation and near‐complete EMT, while the adjacent non‐fibrotic areas have persisting inflammation and EMT changes which may explain the increase in INPP4A in non‐fibrotic regions of the fibrotic lungs either as an inducer of or a compensatory response during EMT. Further, our work succeeds in generating interesting questions relevant to the field: (1) If INPP4A expression follows a non‐linear pattern, could this be used as a molecular marker to define the disease stage or to decide the eligibility for lung transplantation? (2) If the non‐linear pattern of INPP4A expression specific to IPF only or applies to other non‐IPF ILDs as well? (3) In light of the recent studies showing that molecular changes precede histological changes in IPF, it would also be interesting to understand how the upregulated INPP4A in the less fibrotic regions contribute to the IPF disease progression? (4) Other than extent of fibrosis, are there any other gene regulatory mechanisms that control the spatial and temporal expression of INPP4A in IPF? We conclude by saying that the expression of INPP4A in IPF is intricately regulated and depends on several factors including the cellular state, fibrotic heterogeneity of the lung region sampled and the stage of disease progression. Therefore, single cell along with spatial transcriptomics data are required to gain insights into the mechanistic understanding of the role of INPP4A in the IPF pathophysiology.

## AUTHOR CONTRIBUTIONS

M.A. conceived the study, designed and performed the experiments, analyzed the data, interpreted results of experiments, prepared figures and wrote the manuscript. B.P. designed and performed the experiments, prepared figures. A.G. performed the experiments. M.S. assisted in data analysis. Y.S.P. provided human IPF samples. A.A. provided infrastructural support, interpreted results of experiments, edited and revised the manuscript and supervised the overall study. N.K.B. performed the experiments, analyzed the data, prepared figures, assisted in manuscript writing, edited and revised the manuscript. All the authors approved the final version of manuscript.

## FUNDING INFORMATION

This work was mainly supported by Welcome Trust, Grant (IA/CPHS/14/1/501489) to A.A. Y.S.P. is supported by grants NIH R01 HL088029 and R01 HL142061. N.K.B. acknowledges financial support in the form of fellowship received as a senior research associate from the Council of Scientific and Industrial Research, (CSIR). M.A. acknowledges fellowship and contingency support as a junior and senior research fellow from the University Grants Commission (UGC) and as a senior research fellow from Indian Council of Medical Research (ICMR).

## CONFLICT OF INTEREST STATEMENT

The authors declare no competing interests.

## ETHICS STATEMENT

Lung samples were obtained incidental to patient thoracic surgery at Mayo Clinic Rochester via IRB‐approved protocols. Patient consent was obtained during pre‐surgical evaluation (Pulmonary, Oncology, or Thoracic Surgery Clinics). All potential patients were reviewed and consented prior to surgery in non‐threatening environs. Eighteen months old male C57BL/6 mice were maintained at CSIR‐Institute of Genomics and Integrative Biology (IGIB), Delhi, India. The institutional animal ethics committee had approved all protocols used.

## Supporting information


Figure S1.

Figure S2.

Figure S3.

Figure S4.

Figure S5.

Figure S6.

Figure S7.
