# Supplementary figures and images for: Plausible role of INPP4A dysregulation in idiopathic pulmonary fibrosis
Source: Physiol Rep. 2024 May 8;12(9):e16032. doi: 10.14814/phy2.16032 (PMC11078778; doi:10.14814/phy2.16032)

A

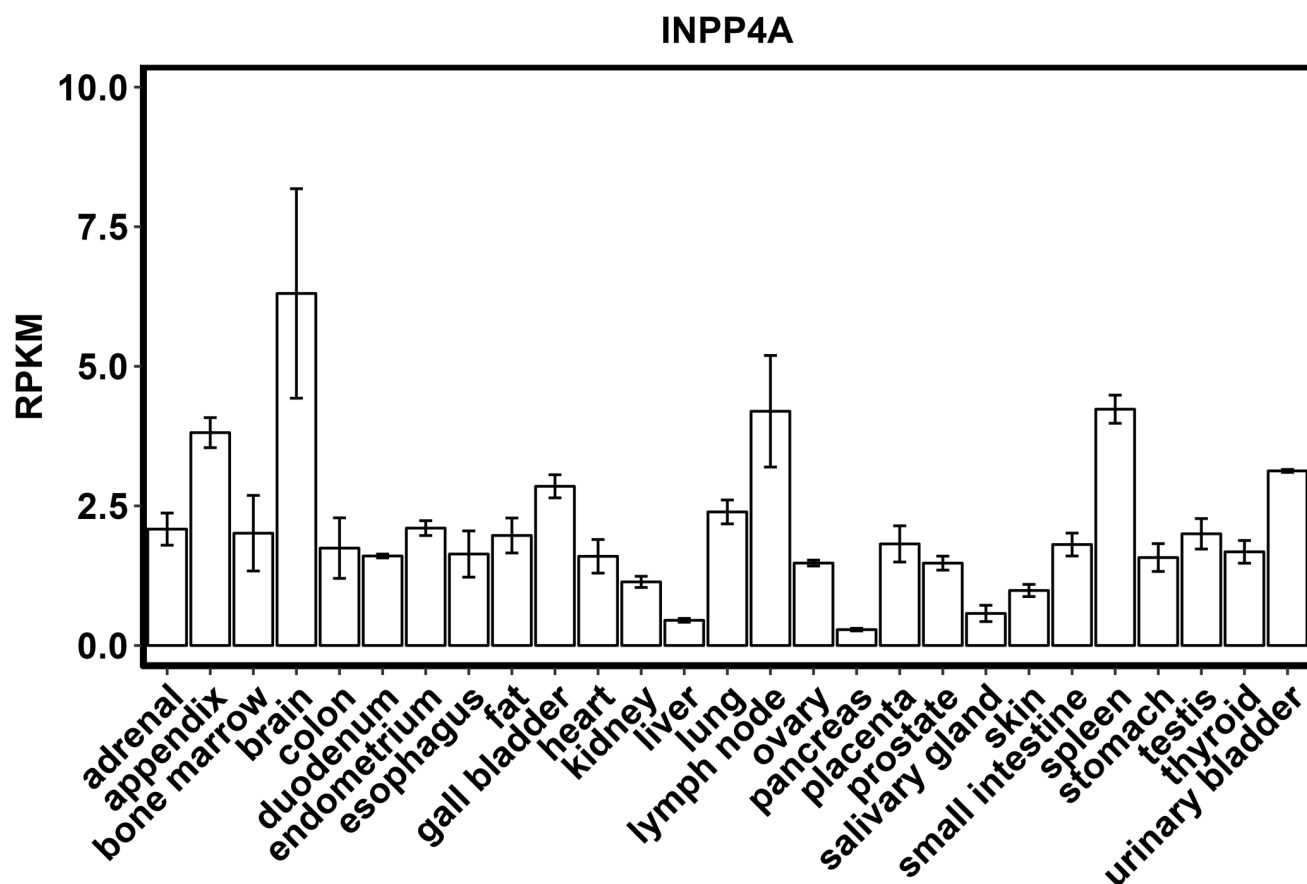

B

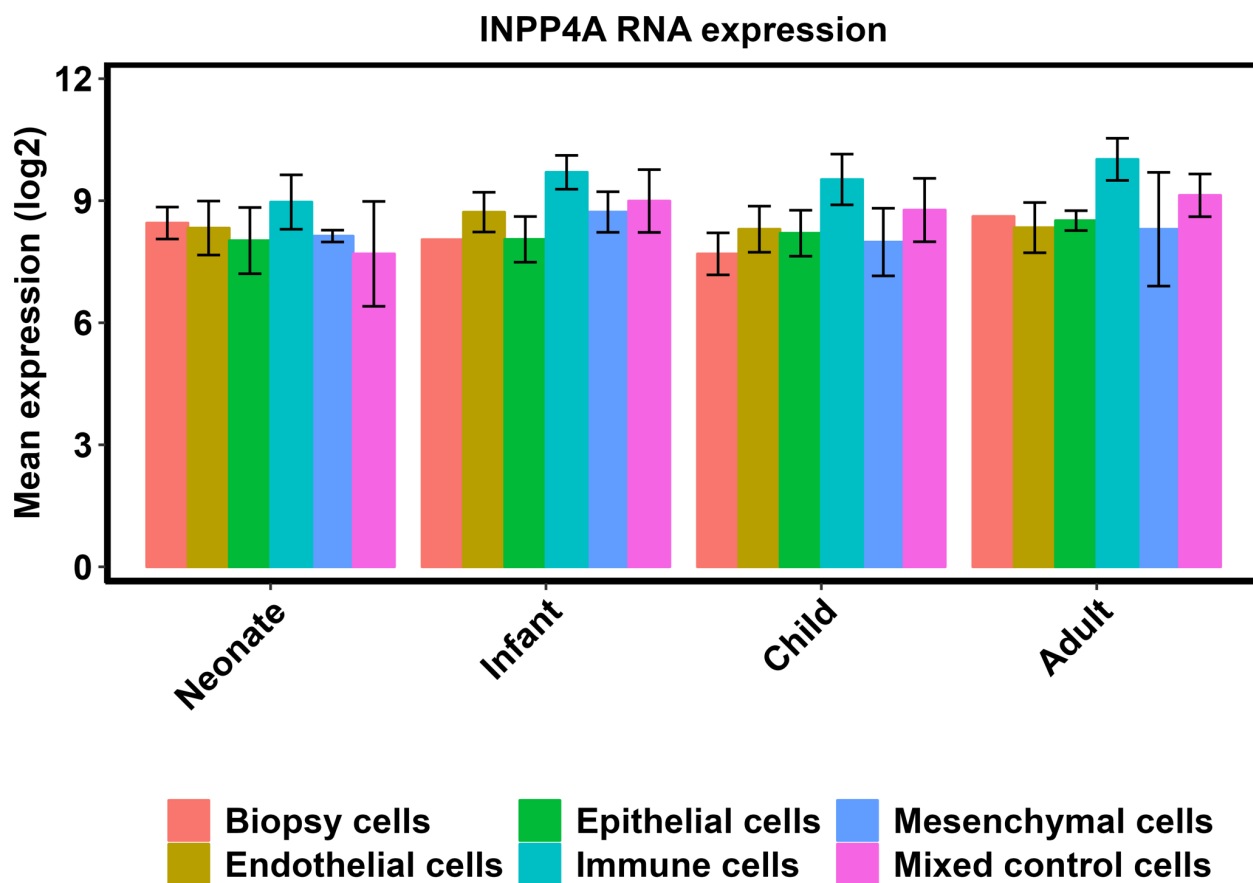

C

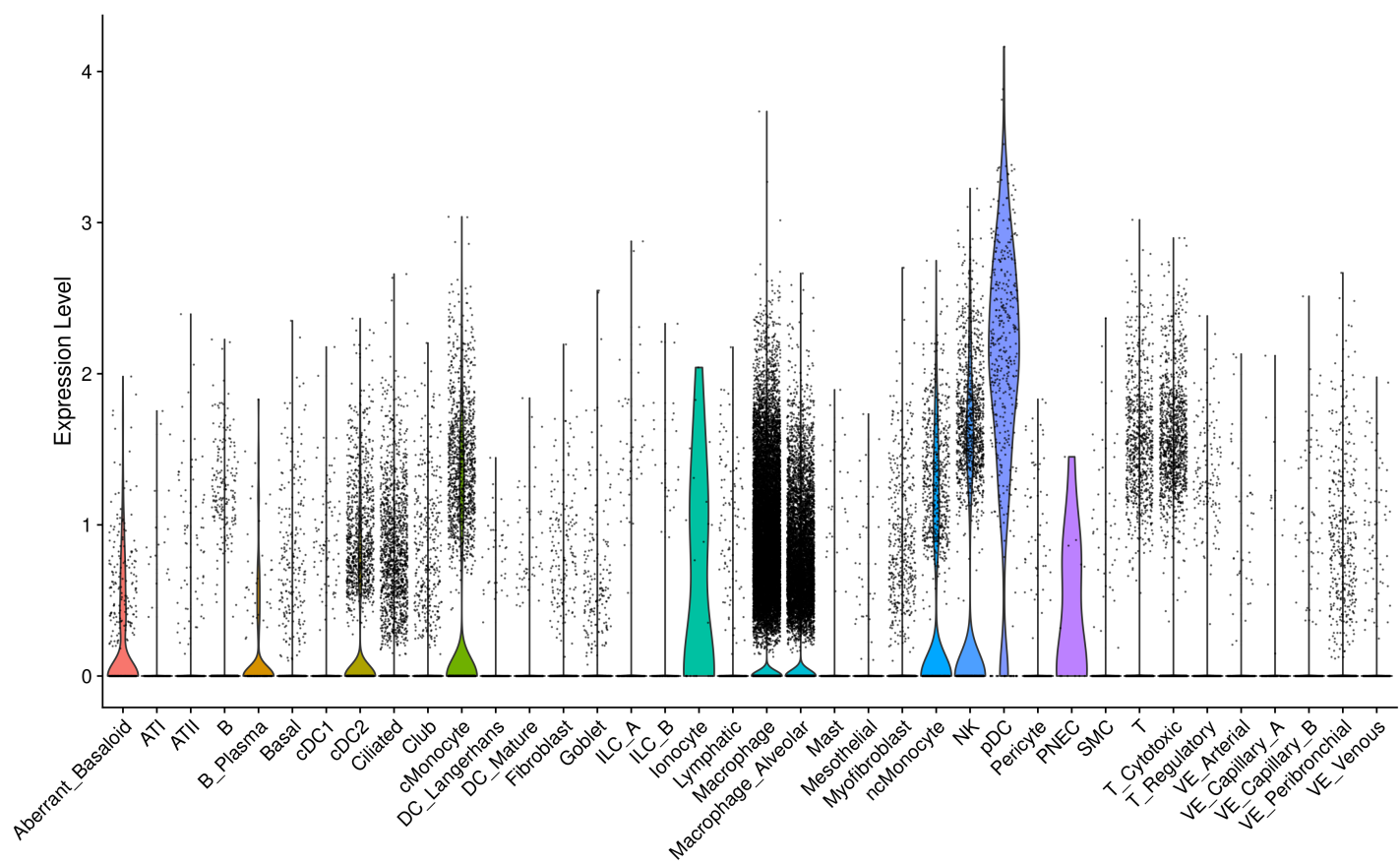

Supplement: Supplementary file 1 — Figure S1. [file PHY2-12-e16032-s001.pdf]

**A**

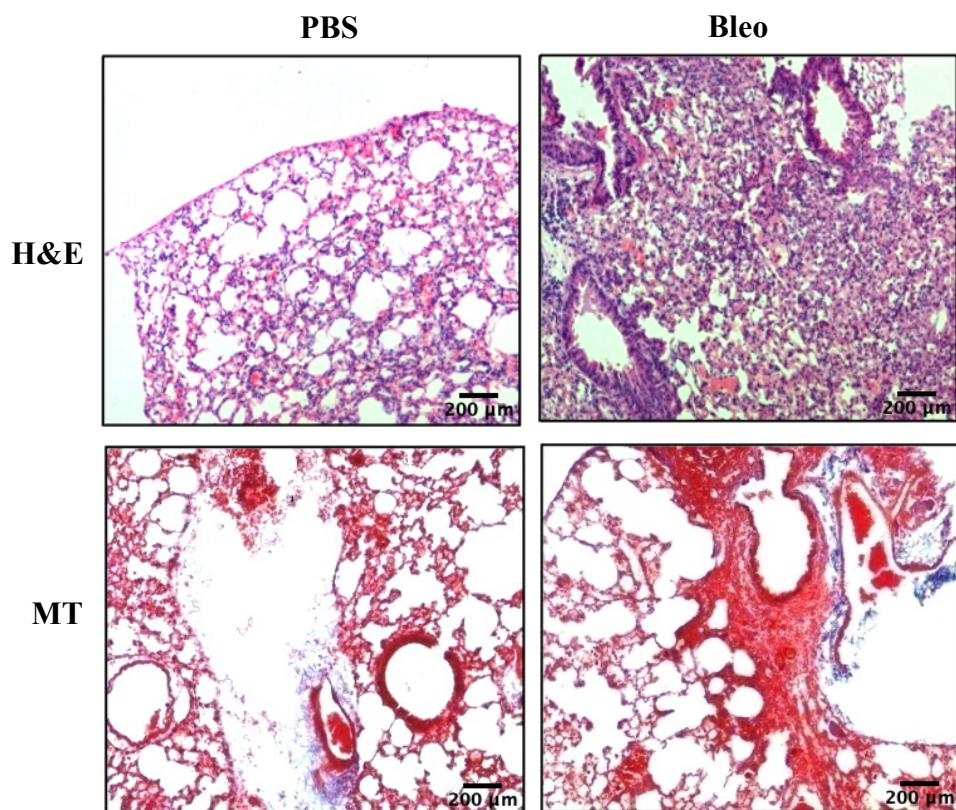

**B**

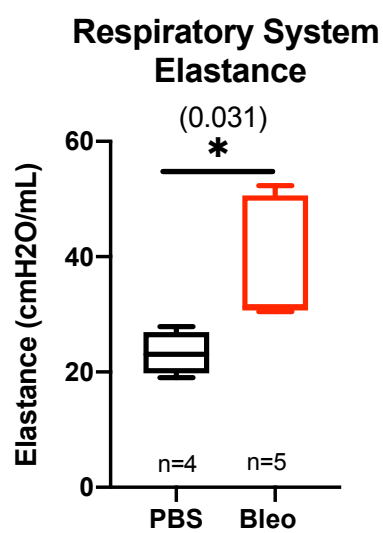

**C**

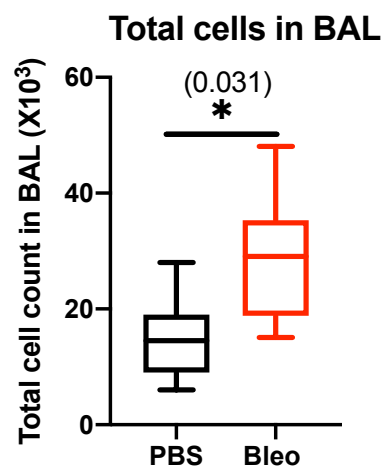

**D**

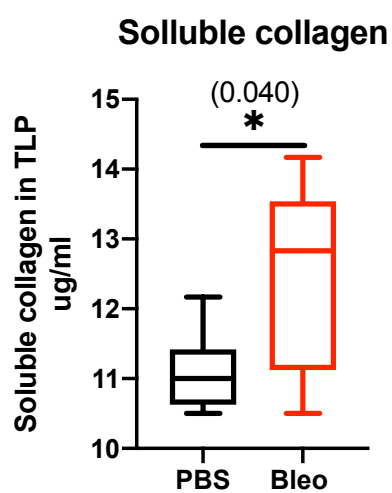

**E**

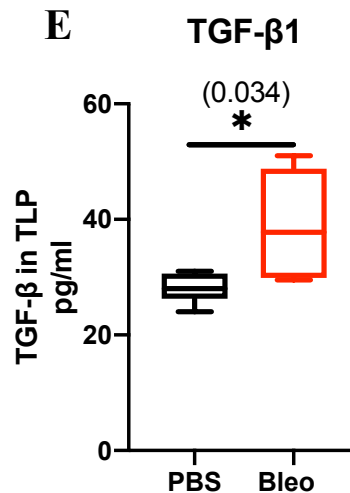

Supplement: Supplementary file 2 — Figure S2. [file PHY2-12-e16032-s007.pdf]

**A**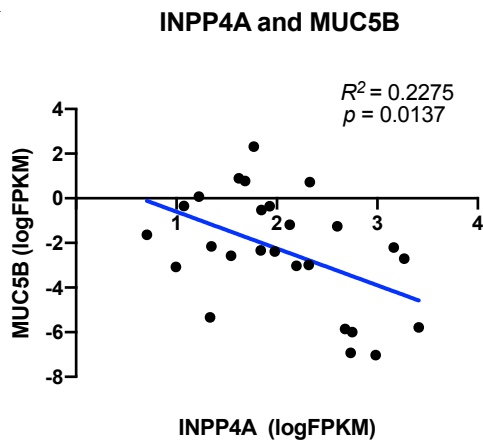**B**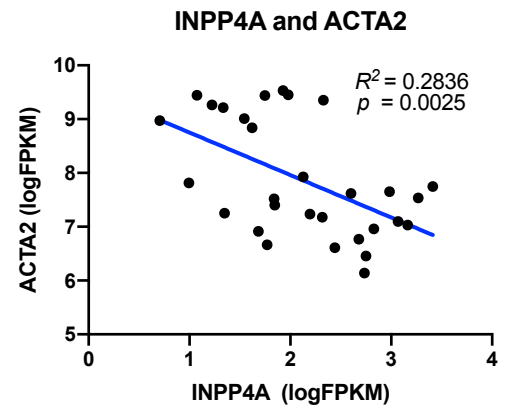**C**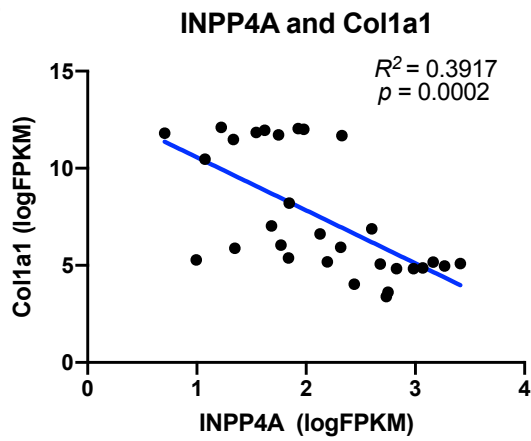**D**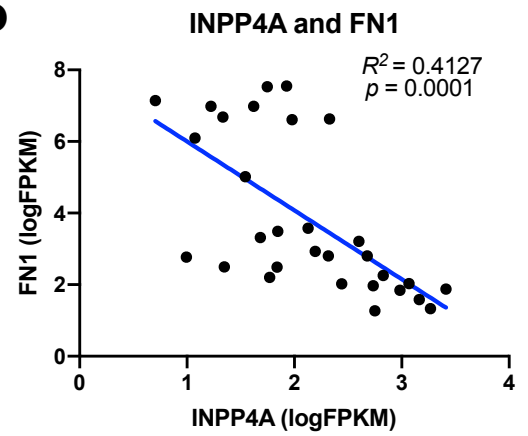**E**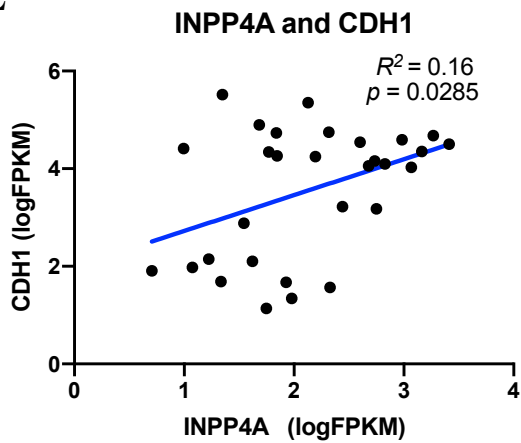**F**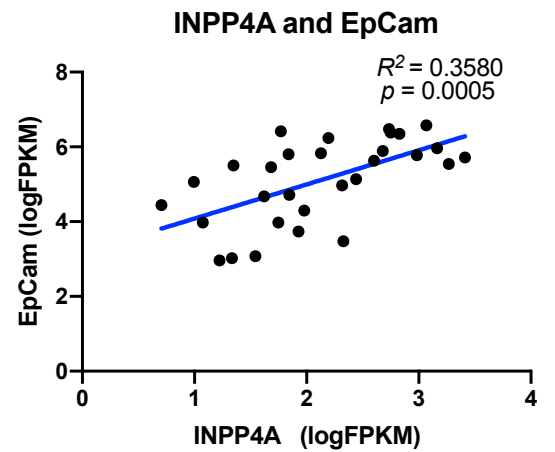**G**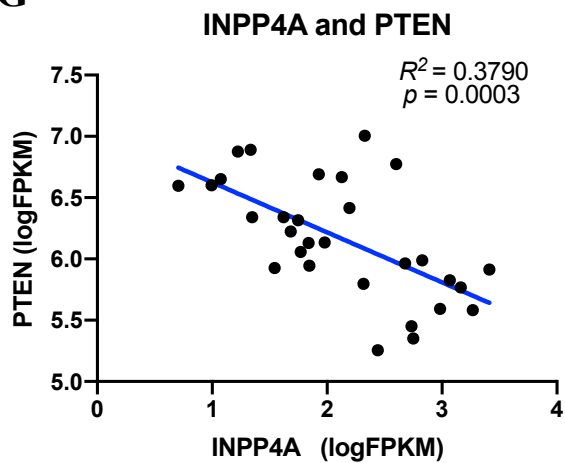

Supplement: Supplementary file 3 — Figure S3. [file PHY2-12-e16032-s004.pdf]

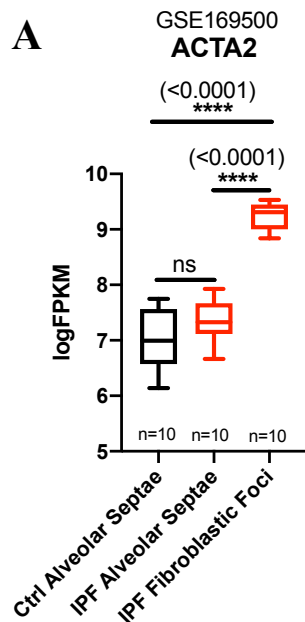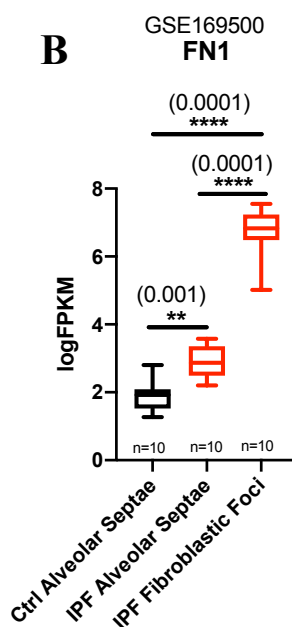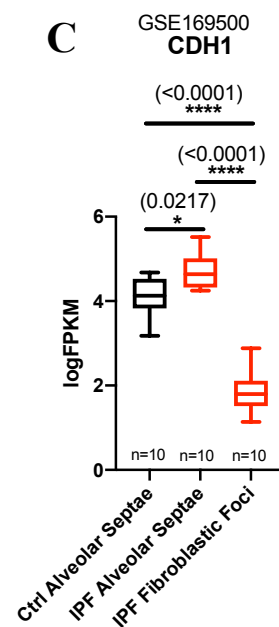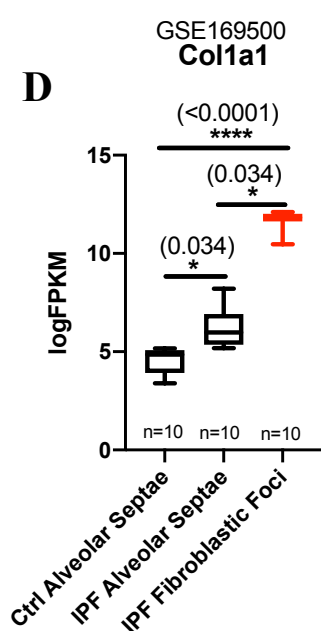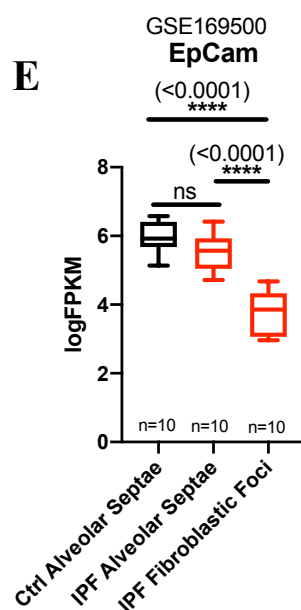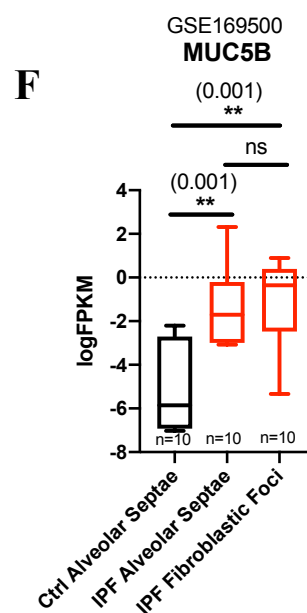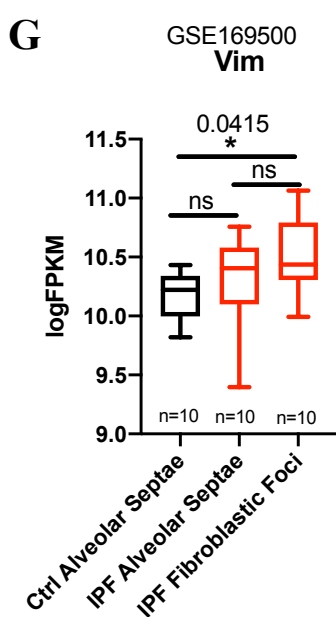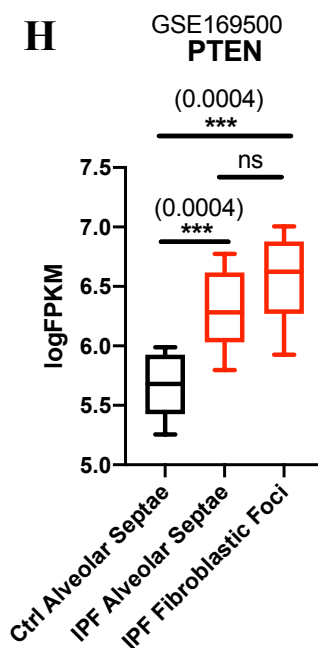

Supplement: Supplementary file 4 — Figure S4. [file PHY2-12-e16032-s002.pdf]

**Phase Contrast**

**Vehicle**

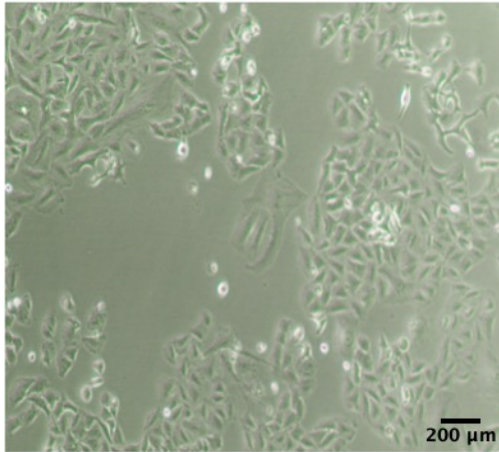

**TGF- $\beta$ 1 (5ng/ml, 120h)**

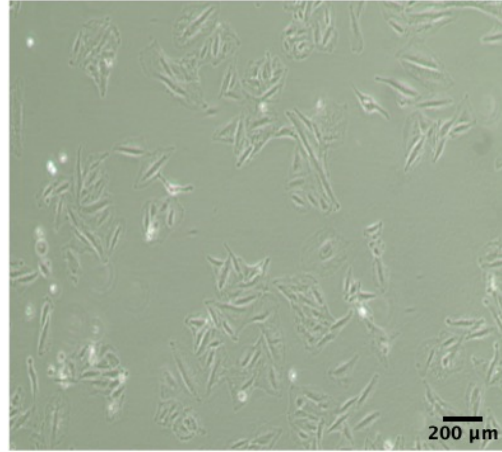

Supplement: Supplementary file 5 — Figure S5. [file PHY2-12-e16032-s006.pdf]

**A**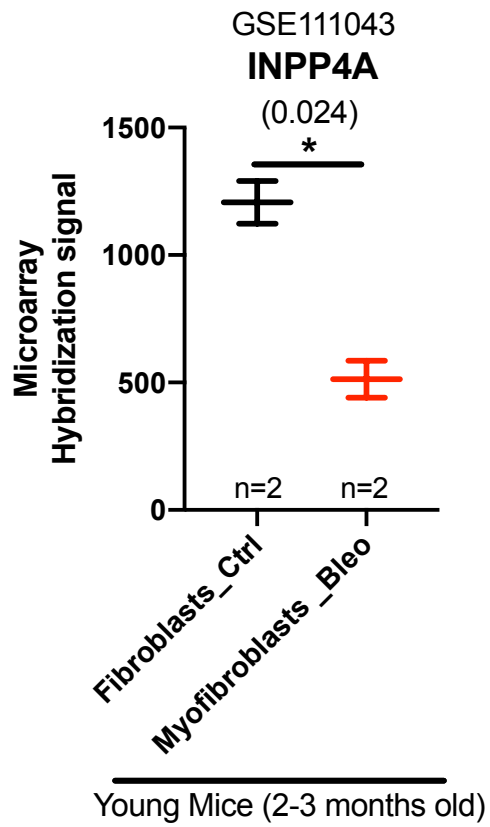**B**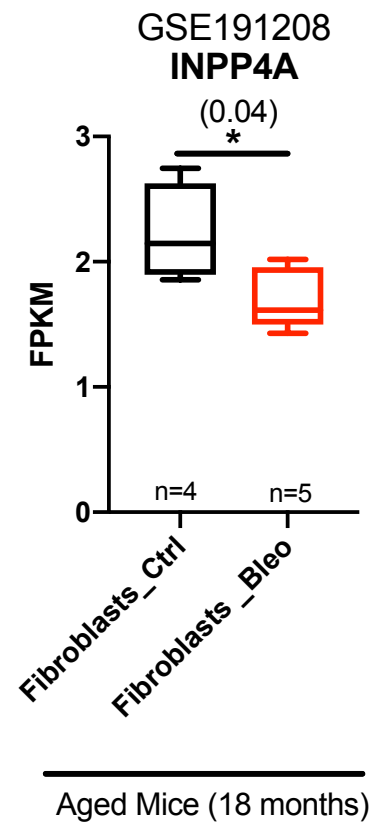

Supplement: Supplementary file 6 — Figure S6. [file PHY2-12-e16032-s005.pdf]

A

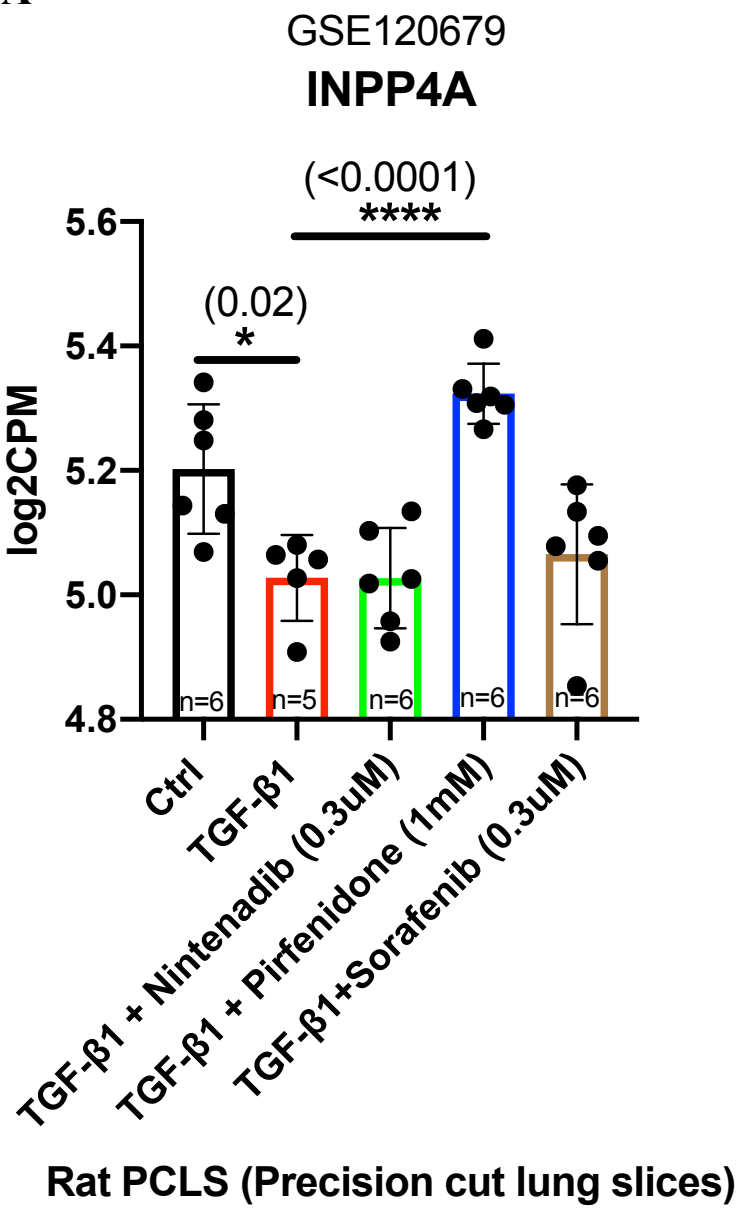

B

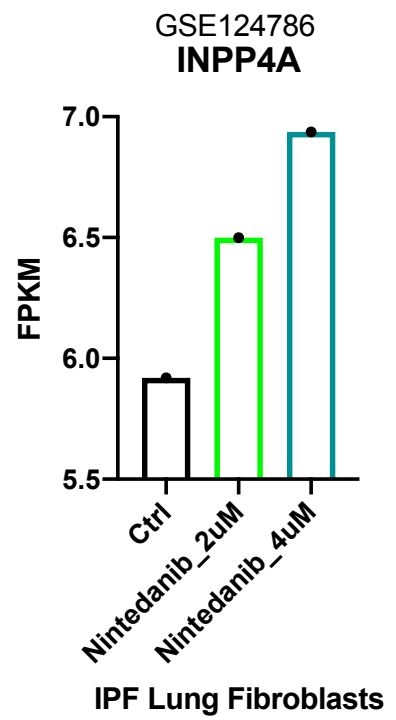

Supplement: Supplementary file 7 — Figure S7. [file PHY2-12-e16032-s003.pdf]
